# Efficacy and safety of apatinib-containing neoadjuvant regimens for resectable gastric and gastroesophageal junction adenocarcinoma: a systematic review and single-arm meta-analysis

**DOI:** 10.3389/fonc.2026.1910490

**Published:** 2026-10-19

**Authors:** Kaiyi Xin, Zhilu Chen, Liu Yang, Li Qin, Shu Chen, Tianjiao Xiao, Yinghao Zhao

**Affiliations:** 1 Department of Thoracic Surgery, The Second Hospital of Jilin University, Changchun, China; 2 Department of Gastrointestinal Medicine, The First Hospital of Jilin University, Changchun, China

**Keywords:** combination therapy, efficacy, gastric/gastroesophageal junction tumor, neoadjuvant apatinib, safety, single-arm meta-analysis

## Abstract

**Background:**

Perioperative chemotherapy remains the standard-of-care for resectable gastric and gastroesophageal junction (G/GEJ) adenocarcinoma, yet pathologic complete response (pCR) rates remain low and long-term survival is suboptimal. Apatinib has shown efficacy in advanced G/GEJ cancer, but its role and optimal combination regimens in the neoadjuvant setting remain unclear, and no quantitative evidence synthesis has been published. This systematic review and single-arm meta-analysis aimed to evaluate the efficacy and safety of neoadjuvant apatinib-based regimens for resectable G/GEJ adenocarcinoma.

**Methods:**

A systematic literature search was conducted across PubMed, Embase, the Cochrane Library, Web of Science, and major oncology conference proceedings up to January 1, 2026. Single-arm studies reporting neoadjuvant apatinib-based therapy for resectable G/GEJ adenocarcinoma were included. Primary outcomes were pCR rate, major pathologic response (MPR) rate, R0 resection rate, objective response rate (ORR), and disease control rate (DCR); secondary outcomes were grade ≥3 treatment-related adverse events (TRAEs) and postoperative complications. Pooled proportions with 95% confidence intervals were calculated using logit-transformed single-arm meta-analysis; random- or fixed-effects models were selected based on between-study heterogeneity (I²). Analyses were performed in R, Review Manager 5.4, and Stata 17.0.

**Results:**

Twenty studies enrolling 1026 patients with resectable G/GEJ cancer were initially included. After excluding one potentially overlapping cohort (Jianxian Lin 2024, n=51), 19 studies with 975 patients were included in the primary analysis. The pooled pCR rate of neoadjuvant apatinib-based therapy was 21% (95% CI: 18%–25%, I² = 19.4%), and the pooled major pathologic response (MPR) rate was 39% (95% CI: 34%–45%, I² = 37.8%). The R0 resection rate reached 93% (95% CI: 90%–95%, I² = 37.6%), with an objective response rate (ORR) of 70% (95% CI: 66%–74%, I² = 24.4%) and a disease control rate (DCR) of 95% (95% CI: 92%–96%, I² = 0%). The overall incidence of grade ≥3 treatment-related adverse events (TRAEs) was 31% (95% CI: 21%–43%, I² = 80.1%), and the pooled rate of postoperative complications was 27% (95% CI: 22%–32%, I² = 44.6%). In exploratory subgroup analysis, pCR rates were 12% in the nACT group and 22% in the nAICT group, with similar directional differences observed in MPR, ORR, and R0 resection; however, these were indirect, non-randomized comparisons and are presented descriptively as hypothesis-generating.

**Conclusion:**

This meta-analysis reports that neoadjuvant apatinib-containing regimens are associated with a pooled pCR rate of 21%, MPR rate of 39%, R0 resection rate of 93%, and DCR of 95%. These descriptive estimates suggest that apatinib-containing neoadjuvant therapy is feasible in selected patients and warrants further investigation in randomized controlled trials. However, because the evidence is predominantly non-comparative and heterogeneous, no conclusion regarding comparative efficacy or safety superiority over conventional therapy can be drawn.

**Systematic review registration:**

https://www.crd.york.ac.uk/prospero/, identifier CRD42023478481.

## Introduction

Gastric cancer (GC) is a primary epithelial malignant tumor originating in the stomach. It is one of the most common malignant tumors worldwide and ranks as the fourth leading cause of cancer-related deaths (1, 2). Its incidence is related to a variety of factors such as Helicobacter pylori infection, dietary habits, genetic susceptibility, environmental pollution, etc. (3, 4). Gastroesophageal junction (GEJ) tumors are a type of heterogeneous malignant tumor. Their incidence rate is on the rise globally, and it is particularly severe in East Asia. The classification of this tumor mainly uses the Siewert type (including Siewert type I, II, and III) (5–7). While the 5-year overall survival (OS) of patients with early-stage gastric cancer was 93.6%, the 5-year OS of patients with advanced-stage cancer dropped dramatically to 17.9% (8). For now, it remains that surgical resection plus lymph node dissection is the only potentially curative treatment for patients with G/GEJ (9). Compared with surgery alone, neoadjuvant chemotherapy in the MAGIC and FNCLCC/FFCD 9703 trials resulted in R0 resection rates of 79% and 86%, respectively, and improved 5-year overall survival from 23% to 24% to approximately 36%–38% (10, 11). In gastric cancer, perioperative or neoadjuvant chemotherapy increases R0 resection and survival rates and has been recommended by guidelines all over the world (4). Nevertheless, there is no favorable pathologic response associated with overall survival, with a pathologic complete response (pCR) of approximately 6%–16% (12).

At present, neoadjuvant therapy is gaining increasing attention among patients with gastric cancer and tumors in the gastroesophageal junction region due to its promising therapeutic effects and controlled safety profile (13). As a hallmark of cancer, angiogenesis is responsible for the growth and metastasis of aggressive tumors (14). Apatinib, an oral tyrosine kinase inhibitor (TKI) of vascular endothelial growth factor receptor 2 developed in China, has a good efficacy and tolerable safety profile for systemic treatment of advanced gastric cancer (15–18). A previous phase III trial showed that apatinib (versus placebo) improved progression-free survival (PFS) (2.6 months versus 1.8 months) and overall survival (OS) (6.5 months versus 4.7 months) in patients with advanced gastric cancer who were refractory to two or more chemotherapy regimens (19). A study has shown that apatinib can enhance the efficacy of POF (paclitaxel, oxaliplatin, and 5-FU/leucovorin) in treating advanced gastric cancer. The ORR in the high-dose apatinib group was 90.0%, which was significantly higher than 66.7% in the low-dose group (20), supporting further evaluation of apatinib-containing combinations in the neoadjuvant setting.

In recent years, multiple independent research groups have successively reported preliminary clinical data of apatinib-containing neoadjuvant regimens, but the evidence is scattered, and there is still a lack of systematic quantitative synthesis of efficacy and safety. In addition, the optimal combination mode of apatinib in neoadjuvant setting and the comparative efficacy of different regimens are still unclear. Therefore, it is urgent to conduct a systematic review and meta-analysis to integrate existing evidence, clarify the clinical value of neoadjuvant apatinib, and provide reference for clinical decision-making and subsequent trial design.

## Methods

### Data sources and search strategy

For all data included in the current study, it is publicly available in PubMed, Embase, Web of Science and Cochrane library databases. The latest unpublished clinical trials on neoadjuvant apatinib for G/GEJ from the American Society of Clinical Oncology (ASCO), the European Society for Medical Oncology (ESMO), and other international oncology congresses were also included. A search of PubMed, Embase, Web of Science, and International Congress of Oncology databases was performed to identify candidate articles for analysis until January 1, 2026. All keywords were searched through MeSH, mainly gastric cancer, gastroesophageal junction tumors, apatinib, neoadjuvant, and so on. This systematic evaluation and meta-analysis followed the Preferred Reporting Items for Systematic Evaluation and Meta-Analysis (PRISMA) statement (21). This systematic evaluation and meta-analysis are registered with PROSPERO (registration number: CRD42023478481).

### Inclusion and exclusion criteria

In the present meta-analysis, the inclusion criteria were as follows: (1) patients with resectable and histopathologically confirmed G/GEJ; (2) apatinib as the mainstay of neoadjuvant therapy; and (3) reported at least one of the following primary outcomes: pCR, MPR, R0 resection rate, surgical resection rate, incidence of ≥f TRAEs, incidence of surgical complications, CR, PR, SD, and ORR. The exclusion criteria were as follows: (1) patients with unresectable or advanced metastases of G/GEJ; (2) case reports, reviews, or commentaries; (3) multiple articles with overlapping or duplicated data published by different authors; (4) articles that were not written in English; and (5) studies that did not address the key findings of the current meta-analysis.

### Data extraction

Kaiyi Xin and Zhilu Chen, independently completed literature screening and data extraction. Any discrepancies during the process were resolved by consensus discussion, or referred to Yinghao Zhao for final verification and adjudication. The data were recorded using an Excel spreadsheet for archiving. In addition, parameters were extracted in a uniform format, including first author, year of publication, type of study (single arm or RCT), approval number, pathologic typing, pathologic staging, treatment modality, number of enrollees, age, sex ratio, preoperative cycle, TTS (time from final chemotherapy to surgery), postoperative adjuvant cycle, pCR, MPR, TRG0-3, R0 resection rate, surgical resection rate, incidence of ≥f TRAEs, incidence of postoperative complications, CR, PR, ORR, SD, DCR, 1–3-year DFS and 1–3-year OS.

### Quality assessment

The Cochrane 5.1.0 manual was used to evaluate the RCTs included in the study. The MINORS scale is used to evaluate non-randomized controlled trials. Qualitative evaluations were conducted independently by two investigators, and disagreements were resolved by discussion between the two investigators or a third investigator. Possible publication bias in clinical studies was examined using funnel plots and Egger’s test.

### Statistical analysis

All statistical analyses were performed using R software (version 4.3.2; R Foundation for Statistical Computing, Vienna, Austria) with the meta package (version 6.5-0), Review Manager 5.4 (Cochrane Collaboration, Oxford, UK), and Stata 17.0 (StataCorp LLC, College Station, TX, USA). Although nine randomized controlled trials (RCTs) were included, only the apatinib-containing arms were extracted and analyzed as single-arm cohorts, because the present study was designed as a meta-analysis of non-comparative single-arm proportions. No randomized between-arm comparisons were performed, and the Cochrane risk-of-bias assessment applies to the original RCTs rather than to the pooled single-arm estimates. Potentially overlapping cohorts from the same research group (Jianxian Lin 2021, 2024, and 2025) were identified. To avoid double-counting of patients, the Jianxian Lin 2024 cohort (n=51) was excluded from the primary analysis; thus, the primary pooled estimates were based on 19 studies (975 patients). In a sensitivity analysis, this cohort was re-included (20 studies, 1026 patients), and the pooled estimates remained consistent. Given that the majority of included studies were single-arm clinical trials reporting binary outcomes as proportions (e.g., pathological complete response [pCR] rate, R0 resection rate, incidence of adverse events), we conducted a meta-analysis of non-comparative single-arm binary data. To stabilize the variance of event rates—particularly for rates approaching 0 or 1—and to ensure that the confidence intervals remained bounded within the [0,1] interval, the logit transformation was applied to the raw event rates of each individual study. Pooled event rates with corresponding 95% confidence intervals (CIs) were calculated using the inverse-variance weighting method. The estimates were then back-transformed from the logit scale to the original proportion scale to facilitate clinical interpretation. Subgroup analyses were descriptive only. Pooled proportions were calculated separately within each regimen. No formal between-regimen comparative analysis or risk difference was performed, because the estimates were derived from independent single-arm cohorts. Q-test *p* < 0.05 or I^2^ > 50% considered that there was significant heterogeneity in the literature, and a random effects model was used; otherwise, a fixed effects model was used. In addition, sensitivity analyses were performed by sequentially removing individual studies to assess the stability of the combined results of these studies. For studies where heterogeneity was significant and could not be reduced using sensitivity analyses, further subgroup analyses were performed to explore the sources of heterogeneity. Subgroup analyses across different regimens were exploratory in nature, not intended for confirmatory efficacy comparison; caution should be exercised when interpreting subgroup differences. Publication bias was tested using a funnel plot and Egger’s regression test. P values for overall effect were not used to infer treatment efficacy. In single-arm meta-analyses, such P values test whether a pooled proportion differs from zero and do not provide evidence of treatment benefit in the absence of a comparator. Therefore, pooled proportions are reported with 95% CIs and heterogeneity statistics.

## Results

### The characteristics of the included studies

As shown in **Figure 1**, the PRISMA diagram represents the research selection process (22). According to the search strategy, a total of 483 articles were identified. A total of 20 studies involving 1026 patients met the inclusion criteria. To identify potentially overlapping cohorts, we cross-referenced trial registration numbers, institution names, and patient enrollment periods across all included studies. Two cohorts from the same research group (Jianxian Lin 2024, and 2025) were identified as potentially overlapping based on shared NCT04195828 registration and overlapping patient enrollment. After excluding one potentially overlapping cohort (Jianxian Lin 2024, n=51), 19 studies with 975 patients were included in the primary analysis. Among the 19 included studies, 3 (23–25) were ongoing trials, so only abstracts could be obtained. The meta-analysis included 11 (24–34) single-arm cohort studies, 7 (23, 35–40) two-arm randomized controlled trials, and 1 (41) three-arm randomized controlled trial. The two randomized controlled studies (39, 41) included neoadjuvant apatinib combined with chemotherapy (nACT) and neoadjuvant apatinib combined with immunotherapy and chemotherapy (nAICT), with 10 study arms receiving nACT, 10 receiving nAICT, and 1 receiving nACRT. Each patient received ≥ 2 courses of neoadjuvant treatment. Most surgical resections were performed 3–5 weeks after neoadjuvant treatment. The main characteristics of the included studies are shown in **Table 1**; the main outcomes are shown in **Tables 2**, **3**. **Table 4**, **Figures 2**, **3** show the overall low to moderate risk of bias of the included studies.

**Figure 1:**
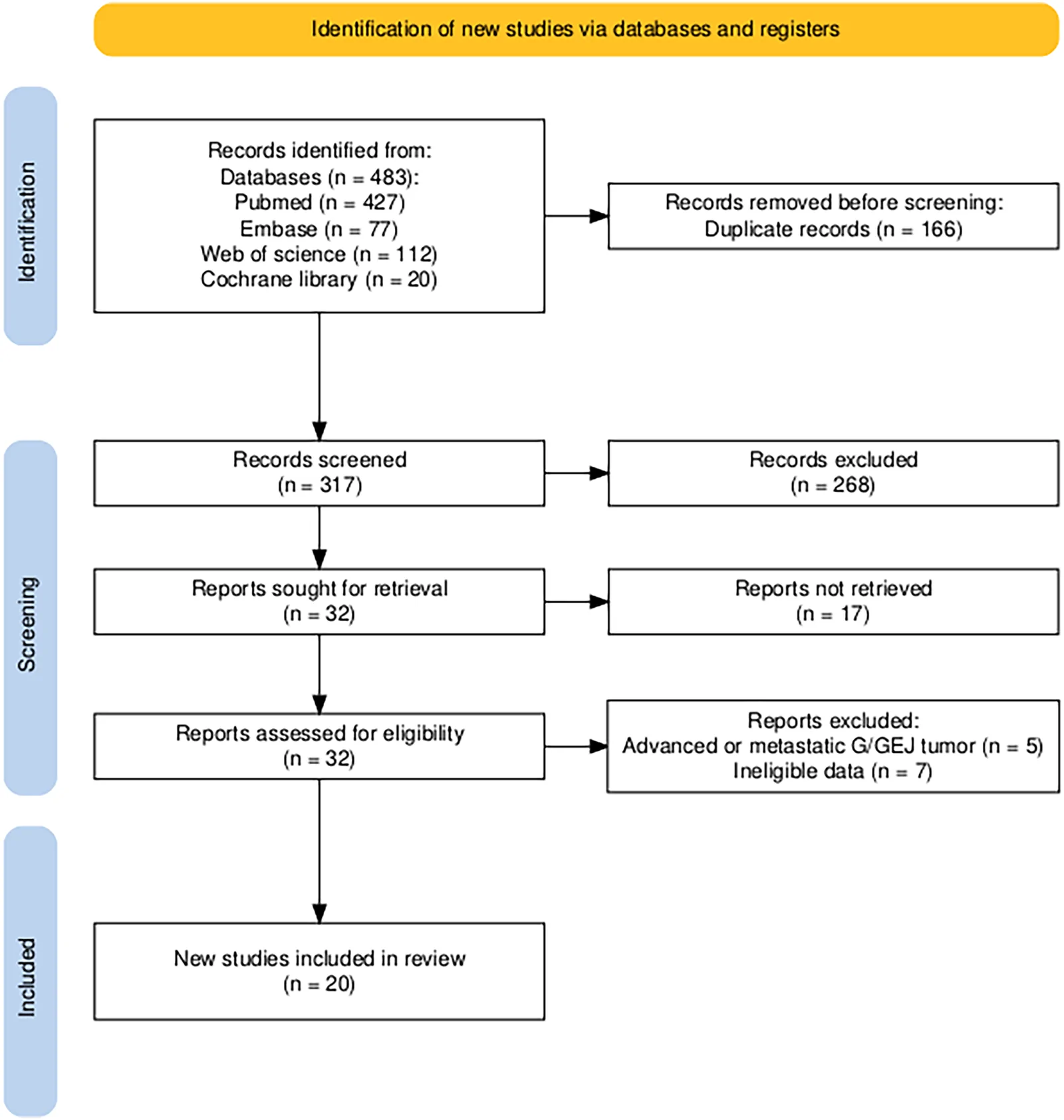
PRISMA flow diagram of study selection. The diagram illustrates the four-phase study selection process: identification, screening, eligibility, and inclusion. A total of 483 records were initially identified from PubMed (n = 427), Embase (n = 77), Web of Science (n = 112), and the Cochrane Library (n = 20). After removing 166 duplicates, 317 records were screened by title and abstract, of which 268 were excluded. Thirty-two full-text articles were assessed for eligibility; 17 were not retrieved and 12 were excluded (5 for advanced/metastatic disease and 7 for ineligible data). Ultimately, 20 studies met the inclusion criteria and were included in the meta-analysis.

**Table 1:** Main features included in the studies.

Author year	NCT number	Study design	TYPE	Combination therapy	Clinical stage	No. of patients	Median age	Tumor type	Preoperative cycle	TTS (W)	Preoperative PD-L1 CPS	dMMR/MSI-H	Her2	The dosage of apatinib(mg/d)
Jian-Xian Lin 2021	NCT03192735	nACT	Single-arm	SOX	T_2-4_N_1-3_	48	63.2	G	2–5	2–4	–	–	–	500
J. Zhang 2020	ChiCTR-IIR-17012911	nACT	RCT	FLOT	–	33	–	LAGC	3	4	–	–	–	–
Yanan Zheng 2020	ChiCTR-OPC-16010061	nACT	Single-arm	SOX	T_3-4a_N_1-3_	29	60	G	2	4	–	–	–	500
Yonglei Zhang 2021	–	nACT	RCT	FLOT	IIB-IV	31	60.19	LAGC	4	2–3	–	–	–	500
Peng-zhe Zhou 2021	–	nACT	RCT	DOS	III	40	65.14	LAGC	2	3–4	–	–	–	850
Zhaoqing Tang 2022	NCT03229096	nACT	Single-arm	CAPOX	T_3-4a_N_1-3_	35	62	G/GEJ	3	3–6	–	–	–	250-500
ZHENFENG WANG 2022	–	nACT	RCT	SOX	T_3-4a_N_1-3_	25	55.2	LAGC	2	3–5	–	–	–	–
Yun Sun 2022	–	nACT	RCT	SOX/CAPOX	T_3-4a_N_1-3_	45	57.2	G/GEJ	3	3–5	–	–	–	375
Fujing Wang/Chunjing Wang 2023	–	nACT	RCT	SOX/CAPOX	T_3-4a_N_1-3_	34	58.9	G	3	–	0 16 1–4 8 5–9 8 ≥10 2	MSI-L/MSS 30 MSI-H 4	–	375
Yun L/Hui Xiong 2023	–	nACT	RCT	SOX/CAPOX	T_3-4a_N_1-3_	50	59.1	G/GEJ	4	–	–	–	–	250
L. Chen 2022	NCT05223088	nAICT	Single-arm	Tislelizumab + SOX	III–IV	25	55.12	G/GEJ	4	–	<1 5 1–5 13 >5 7	MSS 25 MSI-H 0	Negative	250
Song Li 2023	NCT03878472	nAICT	Single-arm	Camrelizumab + SOX	T_4a/b_N_1-3_	25	63	G	2.6	–	–	dMMR/MSI-H 4 pMMR/MSS 21	–	250
Chao Xu 2022		nAICT	Single-arm	PD-1 inhibitor + SOX	T_3-4a_N_1-3_	30	58.2	G	3	3–5	–	–	–	–
H. Zhou 2023	–	nAICT	Single-arm	Sintilimab + FLOT	T_3-4_N_any_	44	56.5	G/GEJ	4	–	–	–	–	–
Chunjing Wang 2023	–	nAICT	RCT	sintilimab/camrelizumab/toripalimab + (SOX)/(CAPOX)	T_3-4a_N_1-3_	39	57.9	G	3	–	1–4 9 5–9 16 ≥10 14	MSI-L/MSS 36 MSI-H 3	–	375
Hui Xiong 2023	–	nAICT	RCT	sintilimab/camrelizumab + (SOX)/(CAPOX)	T_3-4a_N_1-3_	56	58.2	G/GEJ	4	–	–	–	–	250
Tang, Y. 2024	–	nAICT	Single-arm	PD-1 inhibitor + SOX	T_3-4_N_0-3_ M_0_	130	57.4	LAGC	2-3	–	≥1	–	–	500
Zhou, H. 2024	ChiCTR2200055269	nAICT	Single-arm	Sintilimab + FLOT	T_3-4_N_0-4_ M_0_	30	62	LAGC	4	3	–	–	–	250
Lin, J. X. 2025	NCT04195828	nAICT	RCT	Camrelizumab nab-Paclitaxel S-1	T_2-4_N_1-3_ M_0_	51	62.6	LAGC	3	2–3	<1 23 ≥1 27 Unknown 1	–	–	250
Zhan, Z. 2025	–	nAICT	Single-arm	ICIs + SOX	–	38	62.4	LAGC	2–6	2–4	–	–	–	250
Honghai Guo 2021	–	nACRT	Single-arm	CAPOX + Radiotherapy	T_3-4_N_any_	31	61	GEJ	2	8–12	–	–	Negative	250

nACT: Neoadjuvant apatinib combined with chemotherapy; nAICT: Neoadjuvant apatinib combined with immunotherapy and chemotherapy; nACRT: Neoadjuvant apatinib combined with chemotherapy and radiotherapy.

**Table 2:** Main features included in the studies.

Author year	pCR	MPR	R0 resection rate	Surgical resection rate	Operative complication	TRG0	TRG1	TRG2	TRG3	≥3 TRAEs
Jian-Xian Lin 2021	3/48	12/48	36/40	40/48	7/38	–	–	–	–	16/48
J. Zhang 2020	–	–	19/20	20/33	–	–	–	–	–	11/33
Yanan Zheng 2020	4/29	11/29	28/28	28/29	12/28	–	–	–	–	10/29
Yonglei Zhang 2021	–	–	30/31	31/31	–	3/31	17/31	7/31	4/31	–
Peng-zhe Zhou 2021	–	–	31/40	40/40	7/40	–	–	–	–	–
Zhaoqing Tang 2022	2/32	11/32	31/32	32/35	7/32	–	16/32	13/32	2/32	18/32
ZHENFENG WANG 2022	–	–	23/24	24/25	–	–	–	–	–	–
Yun Sun 2022	10/45	–	43/45	45/45	–	–	–	–	–	–
Fujing Wang/Chunjing Wang 2023	5/32	–	30/32	32/34	–	–	10/47	29/47	8/47	–
Yun L/Hui Xiong 2023	8/47	–	44/47	47/50	–	–	7/32	20/32	5/32	–
L. Chen 2022	6/25	9/25	25/25	25/25	–	–	–	–	–	–
Song Li 2023	3/19	5/19	19/23	23/23	7/20	–	5/19	4/19	10/19	–
Chao Xu 2022	6/30	–	28/30	30/30	–	–	–	–	–	–
H. Zhou 2023	14/44	23/44	44/44	44/44	–	–	–	–	–	–
Chunjing Wang 2023	9/39	–	38/39	39/39	–	–	–	–	–	–
Hui Xiong 2023	15/56	–	53/56	56/56	–	–	–	–	–	–
Tang, Y. 2024	28/130		125/130	130/130	–	26/130	26/130	50/130	28/130	15/130
Zhou, H. 2025	8/28	15/28	28/28	28/30	12/28	–	–	–	–	10/30
Lin, J. X. 2025	8/51	17/51	48/49	49/51	10/51	–	–	–	–	22/51
Zhan, Z. 2025	9/38	18/38	37/38	38/38	8/38	–	–	–	–	7/38
Honghai Guo 2021	10/30	–	28/30	29/30	–	10/30	6/30	9/30	3/30	–

**Table 3:** Main features included in the studies.

Author year	CR	PR	SD	ORR	DCR	1 year-DFS	2 year-DFS	3 year-DFS	1 year-OS	2 year-OS	3 year-OS
Jian-Xian Lin 2021	–	–	–	–	–	–	–	–	–	–	–
J. Zhang 2020	–	–	–	–	–	–	–	–	–	–	–
Yanan Zheng 2020	4/29	19/29	5/29	23/29	28/29	–	–	–	–	–	–
Yonglei Zhang 2021	4/31	21/31	4/31	25/31	29/31	–	–	–	–	–	–
Peng-zhe Zhou 2021	7/40	22/40	8/40	29/40	37/40	–	–	–	–	–	–
Zhaoqing Tang 2022	3/32	22/32	7/32	25/32	32/32	–	–	–	–	–	–
ZHENFENG WANG 2022	1/25	15/25	8/25	16/25	24/25	91.7%	75.2%	75.2%	100%	89.6%	78.4%
Yun Sun 2022	2/45	26/45	15/45	28/45	43/45	100%	85.2%	73.8%	97.8%	90.4%	85.9%
Fujing Wang/Chunjing Wang 2023	1/34	19/34	12/34	20/34	32/34	86.1%	72.8%	49.9%	96.9%	76.7%	70.3%
Yun L/Hui Xiong 2023	1/50	29/50	17/50	30/50	47/50	86.9%	75.3%	63.7%	94%	79.5%	76.1%
L. Chen 2022	–	23/25	2/25	23/25	25/25	–	–	–	–	–	–
Song Li 2023	–	–	–	–	–	–	–	–	–	–	–
Chao Xu 2022	2/30	18/30	10/30	20/30	30/30	96.60%	77.70%	–	96.60%	90.10%	–
H. Zhou 2023	13/44	21/44	10/44	34/44	44/44	–	–	–	–	–	–
Chunjing Wang 2023	2/39	27/39	10/39	29/39	39/39	100%	85.7%	76.1%	100%	96.3%	86.7%
Hui Xiong 2023	3/56	38/56	15/56	41/56	56/56	100%	88.6%	69%	100%	94%	86.2%
Tang, Y. 2024	6/130	87/130	30/130	93/130	123/130	–	–	–	–	–	–
Zhou, H. 2025				24/28	28/28	92.6%	–	–	92.4%	84.4%	–
Lin, J. X. 2025				33/51	49/51	–	–	–	–	–	–
Zhan, Z. 2025	0/38	24/38	14/38	24/38	38/38	94.7%	–	–	–	–	–
Honghai Guo 2021	–	–	–	18/30	29/30	96.7%	88.1%	–	100%	96.6	–

**Table 4:** Methodological index for non-randomized studies.

Study	A clearly stated aim	Inclusion of consecutive patients	Prospective collection of data	Endpoint appropriate to the study aim	Unbiased assessment of endpoints	Follow-up period appropriate to the major endpoint	Loss to follow-up not exceeding 5%	Prospective calculation of the study size	Total score
Jian-Xian Lin 2021	2	2	2	2	0	0	2	1	11
Yanan Zheng 2020	2	2	2	2	0	1	2	1	12
Zhaoqing Tang 2022	2	2	2	2	0	0	2	1	11
L. Chen 2022	2	2	2	2	0	1	2	1	12
Song Li 2023	2	2	2	2	0	2	2	0	12
Chao Xu 2022	2	2	2	2	0	1	2	1	12
H. Zhou 2023	2	2	2	2	0	2	2	1	13
Tang, Y 2024	2	2	2	2	0	0	2	1	11
Zhou, H 2024	2	2	2	2	0	1	2	1	12
Zhan, Z. 2025	2	2	2	2	0	1	2	1	12
Honghai Guo 2021	2	2	2	2	0	2	2	1	13

**Figure 2:**
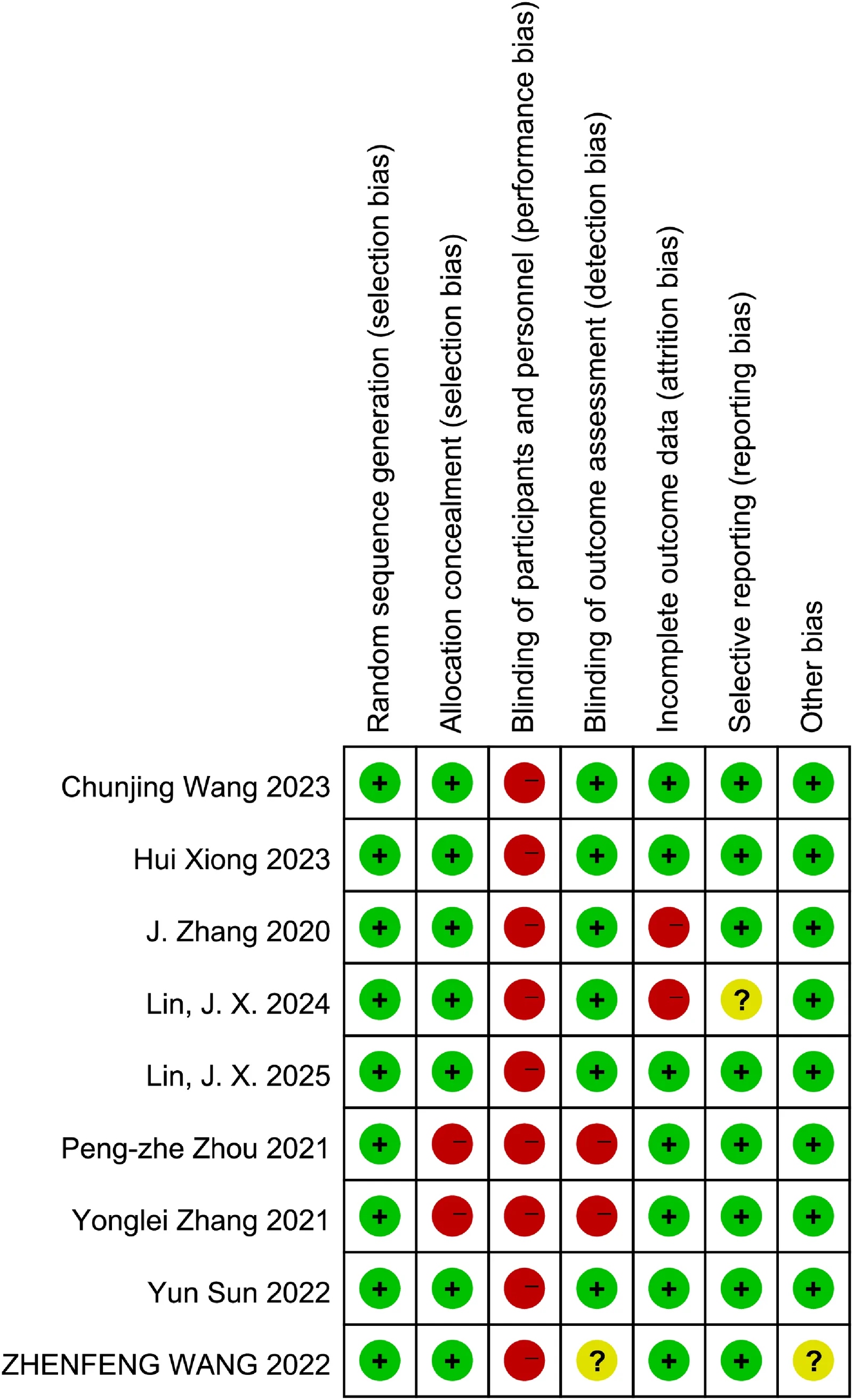
Risk of bias summary for included randomized controlled trials. Each row represents one included RCT (n = 9), and each column represents a domain of the Cochrane Risk of Bias tool: random sequence generation, allocation concealment, blinding of participants and personnel, blinding of outcome assessment, incomplete outcome data, selective reporting, and other bias. Green circles indicate low risk of bias, yellow circles indicate unclear risk, and red circles indicate high risk. Most domains were predominantly low risk, with the exception of blinding of participants and personnel, which was uniformly high risk due to the open-label design of the included trials.

**Figure 3:**
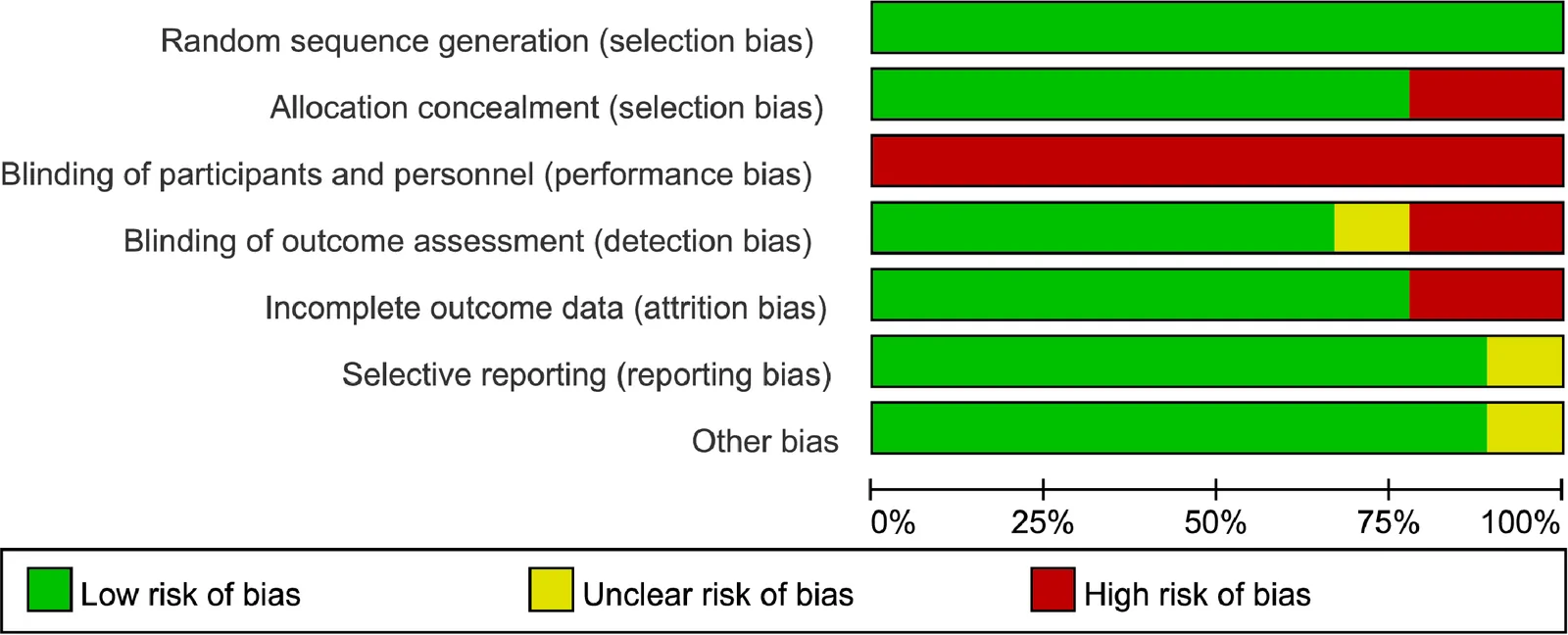
Risk of bias graph for included randomized controlled trials. The stacked bar chart presents the proportion of studies judged as low risk (green), unclear risk (yellow), or high risk (red) across each domain of the Cochrane Risk of Bias tool. The x-axis represents the percentage of studies. Overall, most domains were predominantly low risk, with the exception of blinding of participants and personnel, which was uniformly high risk due to the open-label design of the included trials.

### Risk of bias

The risk of bias for the nine included randomized controlled trials (RCTs) was assessed using the Cochrane Risk of Bias tool (**Figures 2**, **3**). For random sequence generation, all nine RCTs were judged as low risk. For allocation concealment, seven RCTs were low risk and two were high risk. For blinding of participants and personnel (performance bias), all nine RCTs were rated as high risk due to their open-label design, which is inherent to surgical neoadjuvant trials and unlikely to affect objective pathologic outcomes. For blinding of outcome assessment (detection bias), six RCTs were low risk, one was unclear, and two were high risk. For incomplete outcome data (attrition bias), seven RCTs were low risk and two were high risk. For selective reporting, eight RCTs were low risk and one was unclear. For other bias, eight RCTs were low risk and one was unclear. Overall, the included RCTs demonstrated a generally low to moderate risk of bias, with the high-risk ratings predominantly attributable to the unavoidable open-label design.

For the 11 non-randomized studies, methodological quality was evaluated using the MINORS scale (**Table 4**). The total MINORS scores ranged from 11 to 13 out of a maximum of 16, indicating generally moderate-to-good methodological quality. All studies clearly stated their aim, included consecutive patients, and prospectively collected data. The most common limitation was the absence of a prospective sample size calculation and unbiased endpoint assessment. Collectively, the risk of bias assessment supports the overall reliability of the included studies, while acknowledging the inherent limitations of predominantly single-arm, open-label designs.

### Evaluation of efficacy outcomes

#### Pathological response

This study evaluated the efficacy of neoadjuvant apatinib treatment using the pCR rate and MPR rate. Among all the included studies, the pCR rate ranged from 6.3% to 33.3%. In 17 studies that met the criteria, the pooled pCR was 0.21 (95% CI, 0.18–0.25). Using a random-effects model, no significant heterogeneity was detected among the 17 studies (I² = 19.4%, p for heterogeneity = 0.2265; **Figure 4A**).

**Figure 4:**
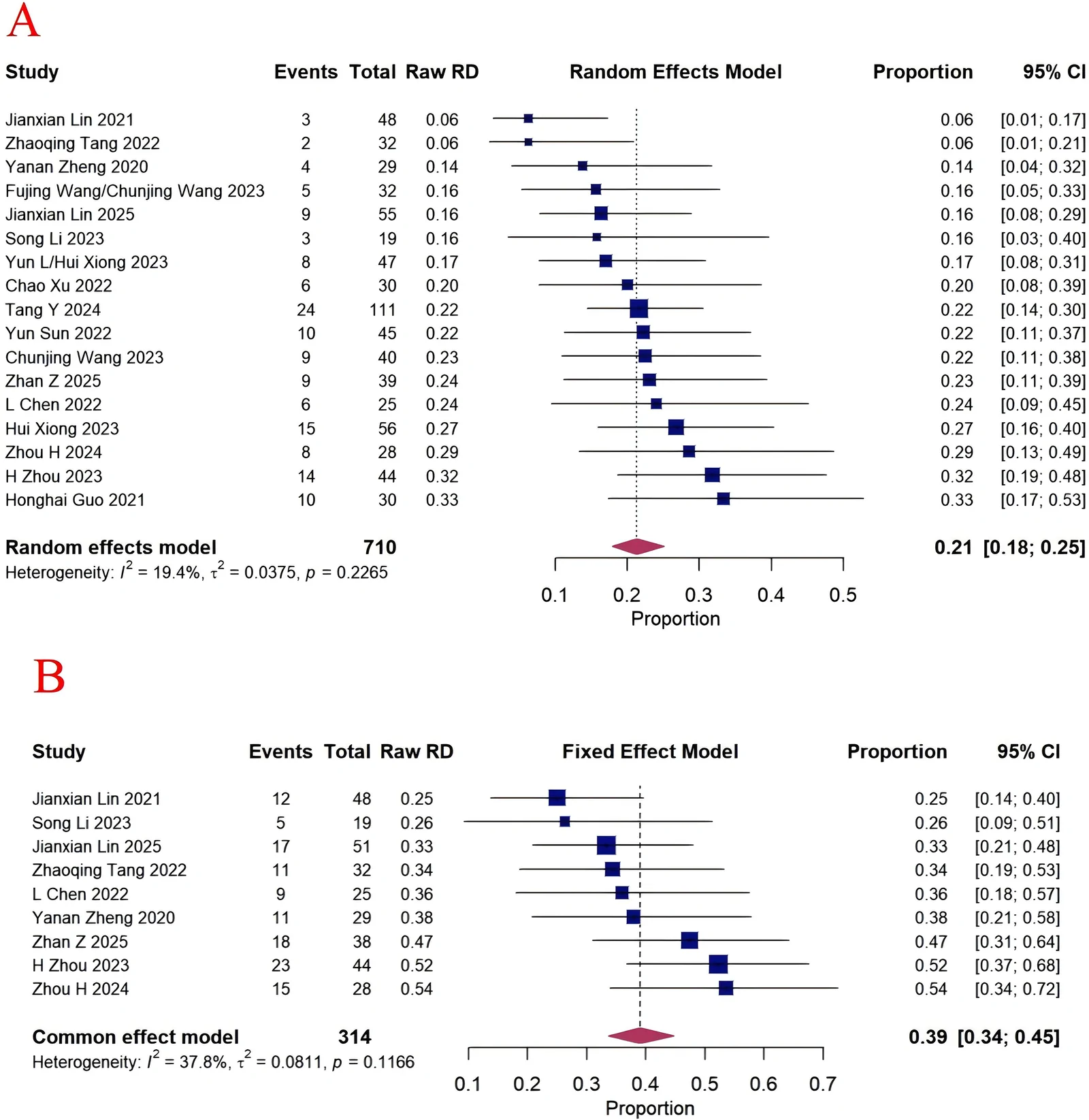
Forest plots of pathologic response rates. **(A)** Pooled pathologic complete response (pCR) rate across 17 studies using a random-effects model (I² = 19.4%, p = 0.2265). The pooled pCR rate was 0.21 (95% CI: 0.18–0.25). **(B)** Pooled major pathologic response (MPR) rate across 9 studies using a fixed-effects model (I² = 37.8%, p = 0.1166). The pooled MPR rate was 0.39 (95% CI: 0.34–0.45). Each square represents the point estimate of an individual study, with horizontal lines indicating 95% confidence intervals; the diamond represents the pooled estimate.

Furthermore, the MPR rates reported in the 9 included studies ranged from 25.0% to 54.0%. The pooled analysis demonstrated a combined MPR rate of 0.39 (95% confidence interval: 0.34–0.45). As no significant heterogeneity was detected among the studies (*p* for heterogeneity = 0.1166, I^2^ = 37.8%), a fixed-effects model was employed for the pooled analysis (**Figure 4B**).

#### Radiological response

The radiological response analysis included varying numbers of studies depending on data availability: CR was reported in 13 studies, PR in 15 studies, SD in 15 studies, ORR in 18 studies, and DCR in 10 studies. The pooled rates were 0.08 (95% CI: 0.05–0.13; I^2^ = 63.4%) for CR, 0.63 (95% CI: 0.58–0.67; I^2^ = 17.6%) for PR, 0.27 (95% CI: 0.23–0.30; I^2^ = 17.3%) for SD, 0.70 (95% CI: 0.66–0.74; I^2^ = 24.4%) for ORR, and 0.95 (95% CI: 0.92–0.96; I^2^ = 0%) for DCR (**Figures 5A–E**).

**Figure 5:**
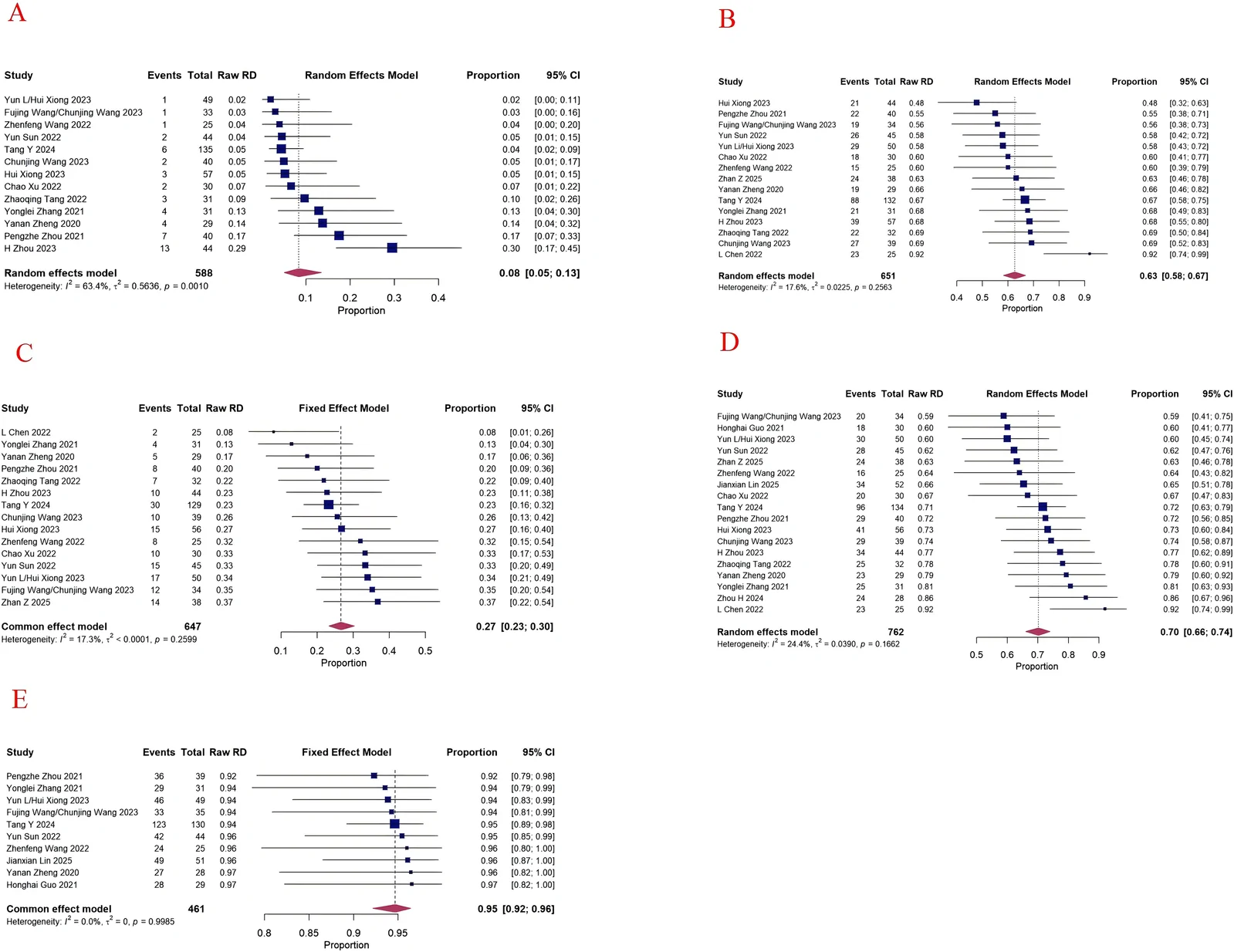
Forest plots of radiological response rates. Pooled rates of **(A)** complete response (CR, n = 13 studies, random-effects model, I² = 63.4%, p = 0.0010), **(B)** partial response (PR, n = 15, random-effects model, I² = 17.6%, p = 0.2563), **(C)** stable disease (SD, n = 15, fixed-effects model, I² = 17.3%, p = 0.2599), **(D)** objective response rate (ORR = CR + PR, n = 18, random-effects model, I² = 24.4%, p = 0.1662), and **(E)** disease control rate (DCR = CR + PR + SD, n = 10, fixed-effects model, I² = 0%, p = 0.9985). The pooled estimates were 0.08 (95% CI: 0.05–0.13), 0.63 (95% CI: 0.58–0.67), 0.27 (95% CI: 0.23–0.30), 0.70 (95% CI: 0.66–0.74), and 0.95 (95% CI: 0.92–0.96), respectively. Each square represents the point estimate of an individual study, with horizontal lines indicating 95% confidence intervals; the diamond represents the pooled estimate.

#### R0 resection rate and surgical resection rate

Among the 19 included studies, four reported a 100% R0 resection rate. The pooled R0 resection rate across the 19 studies was 0.93 (95% CI: 0.90–0.95; I2 = 37.6%, p for heterogeneity = 0.0590; **Figure 6A**). Separately, 8 studies achieved a 100% surgical resection rate; the pooled surgical resection rate was 0.92 (95% CI: 0.84–0.96; I2 = 71.4%, p for heterogeneity = 0.0003; **Figure 6B**).

**Figure 6:**
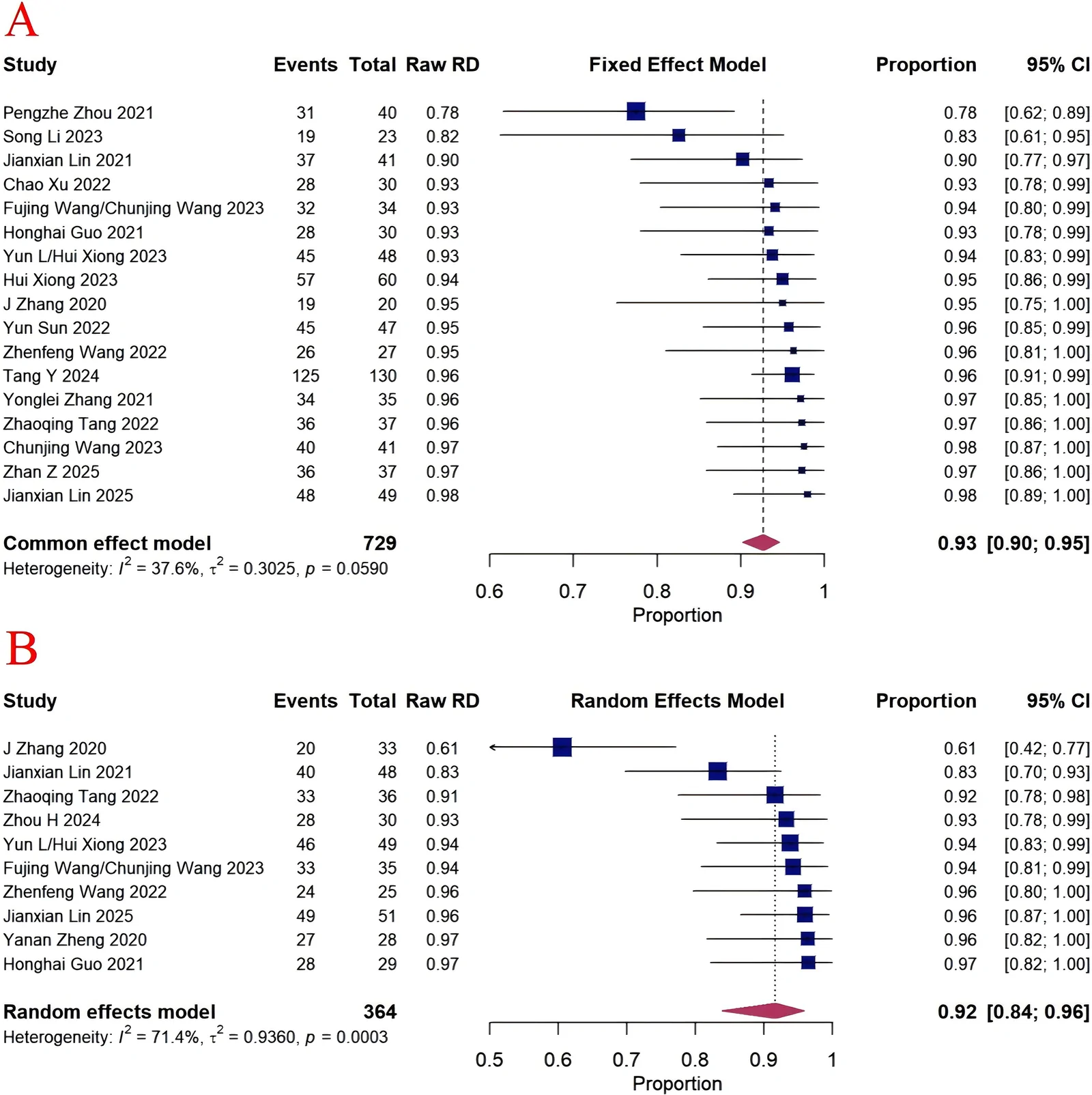
Forest plots of surgical resection rates. **(A)** Pooled R0 resection rate across 19 studies (I² = 37.6%, p = 0.0590), with a reported pooled estimate of 0.93 (95% CI: 0.90–0.95). **(B)** Pooled surgical resection rate across 8 studies using a random-effects model (I² = 71.4%, p = 0.0003), with a pooled estimate of 0.92 (95% CI: 0.84–0.96). Each square represents the point estimate of an individual study, with horizontal lines indicating 95% confidence intervals; the diamond represents the pooled estimate.

### Evaluation of safety outcomes

#### Incidence of ≥f TRAEs

Safety was evaluated as the incidence of grade 3–5 treatment-related adverse events (TRAEs), graded according to the National Cancer Institute Common Terminology Criteria for Adverse Events (NCI-CTCAE, version 4.0). Among the included clinical studies, a total of 8 reported the incidence of grade 3 or higher treatment-related adverse events (TRAEs), involving 392 patients. The pooled incidence of grade ≥r TRAEs was 0.31 (95% CI: 0.21–0.43, I^2^ = 80.1%; **Figure 7A**). No patients died as a result of grade ≥r TRAEs, and surgery was delayed in only 1 patient (93 days) because of treatment-associated pneumonia ^29^. Other primarily controllable adverse events, such as neutropenia, leukopenia, decreased hemoglobin, elevated transaminases, anemia, hand-foot syndrome, proteinuria, anorexia, hemorrhage, hypertension, oral mucositis, pain, and thrombocytopenia, do not result in serious adverse outcomes or lead to higher postoperative mortality.

**Figure 7:**
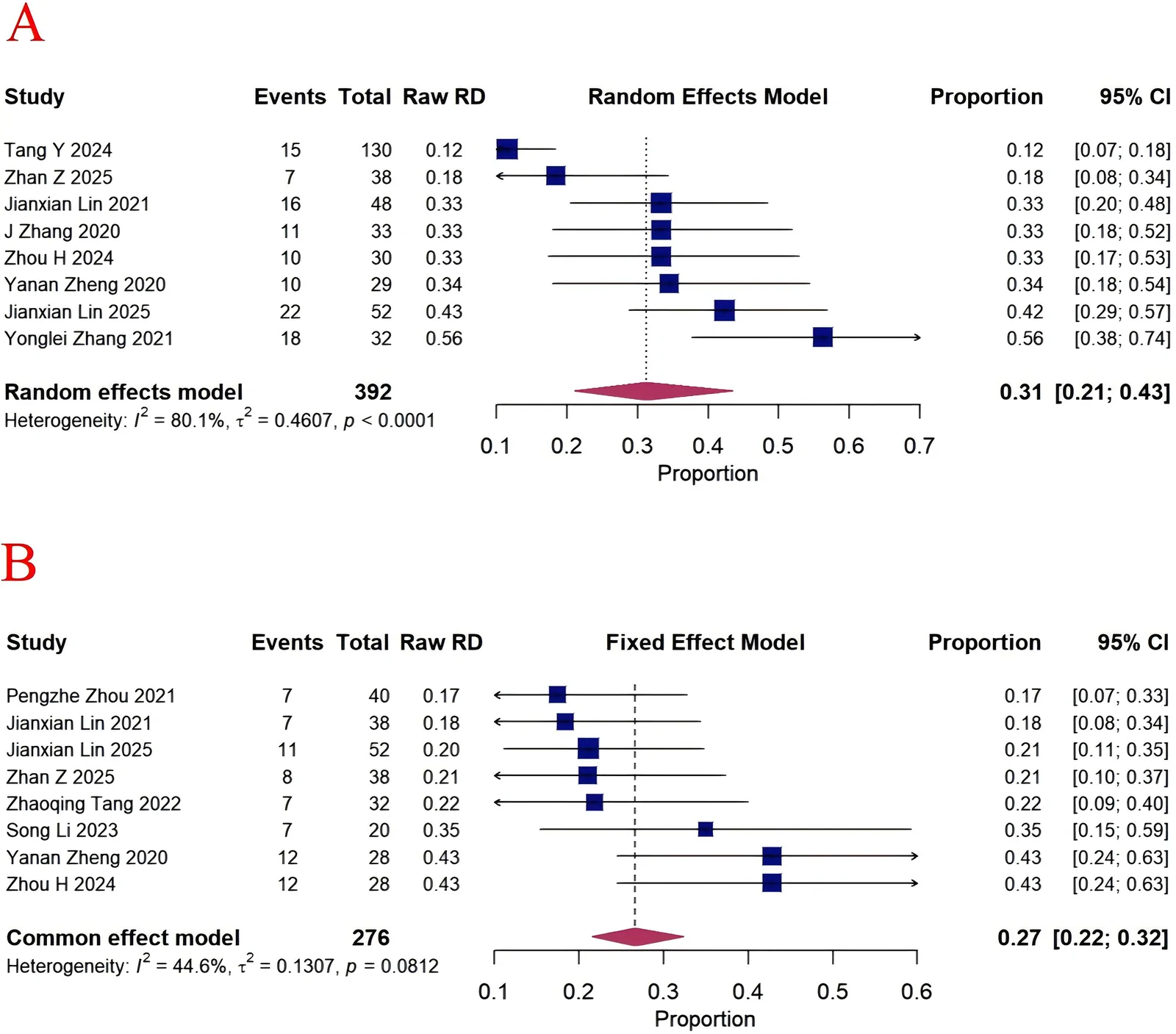
Forest plots of safety outcomes. **(A)** Pooled incidence of grade ≥3 treatment-related adverse events (TRAEs) across 8 studies using a random-effects model (I² = 80.1%, p < 0.0001), with a pooled estimate of 0.31 (95% CI: 0.21–0.43). **(B)** Pooled incidence of postoperative complications across 8 studies using a fixed-effects model (I² = 44.6%, p = 0.0812), with a pooled estimate of 0.27 (95% CI: 0.22–0.32). Each square represents the point estimate of an individual study, with horizontal lines indicating 95% confidence intervals; the diamond represents the pooled estimate.

#### Postoperative complication

Eight trials reported the incidence of postoperative complications, with rates ranging from 17.5% to 43.3%. The pooled incidence of postoperative complications was 0.27 (95% CI: 0.22–0.32, I^2^ = 44.6%; **Figure 7B**). In the included studies, no patient died within 30 days due to postoperative complications. Only one patient underwent a second operation and eventually recovered due to bleeding from the gastrointestinal anastomosis (25). The major postoperative complications included pneumonia, postoperative hemorrhage, abdominal infection, intestinal obstruction, anastomotic leakage, postoperative incisional fatty liquefaction, gastroparesis, pleural effusion, intra-abdominal abscess, pulmonary atelectasis, and respiratory infections, which basically belonged to the Clavien-Dindo class II, and did not have a dramatic impact on the prognosis for patient survival.

#### Sensitivity analysis

Sensitivity analyses were performed to assess the robustness of the pooled estimates. First, to address potential overlap among cohorts from the same research group, the Jianxian Lin 2024 cohort (n=51) was excluded from the primary analysis. When this cohort was re-included in a sensitivity analysis, the pooled estimates and heterogeneity remained essentially unchanged (e.g. pCR: 21% (primary analysis, 19 studies) vs. 19% (sensitivity analysis with Lin 2024 re-included, 20 studies), in **Supplementary Materials**), indicating that the exclusion did not materially affect the results. Second, leave-one-out sensitivity analyses were conducted for the key outcomes (grade ≥g TRAEs, CR, PR, pCR, and surgical resection rate) by sequentially removing each included study. The recalculated pooled effect sizes and their 95% confidence intervals, calculated using R software, showed no substantial change, with widely overlapping intervals before and after each omission, and the direction of effect was consistently maintained (**Figures 8**–**10**). These findings indicate that the pooled results were not unduly driven by any single study. However, given the limited and predominantly non-comparative nature of the available evidence, these results should be interpreted with caution.

**Figure 8:**
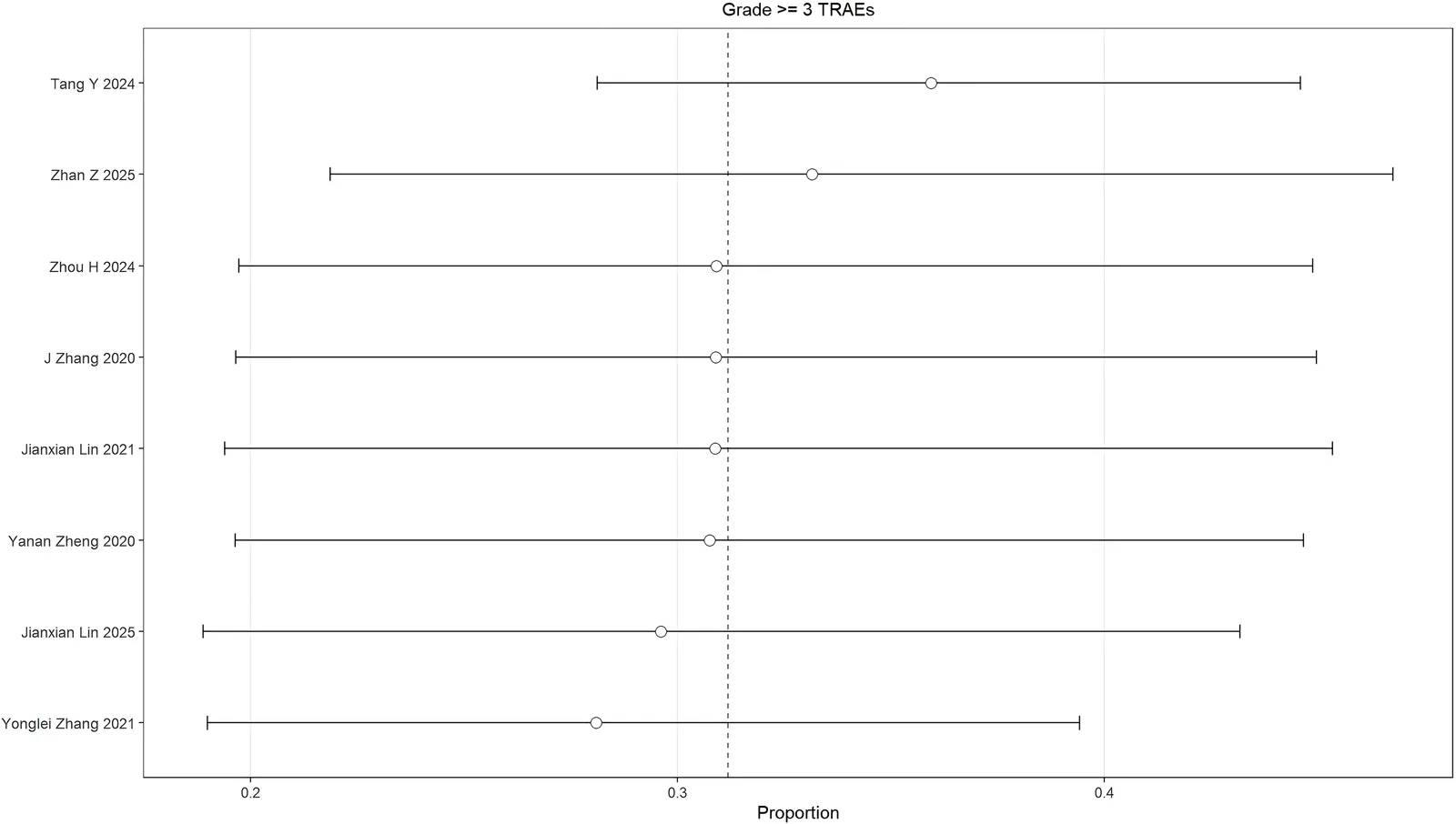
Sensitivity analysis for grade ≥3 TRAEs. Leave-one-out sensitivity analysis was performed by sequentially removing each individual study to assess the stability of the pooled incidence of grade ≥3 TRAEs. The open circles represent the pooled estimate after omitting the indicated study, and the vertical bars represent the corresponding 95% confidence intervals. The results remained consistent across all iterations, indicating that the pooled estimate was not driven by any single study.

**Figure 9:**
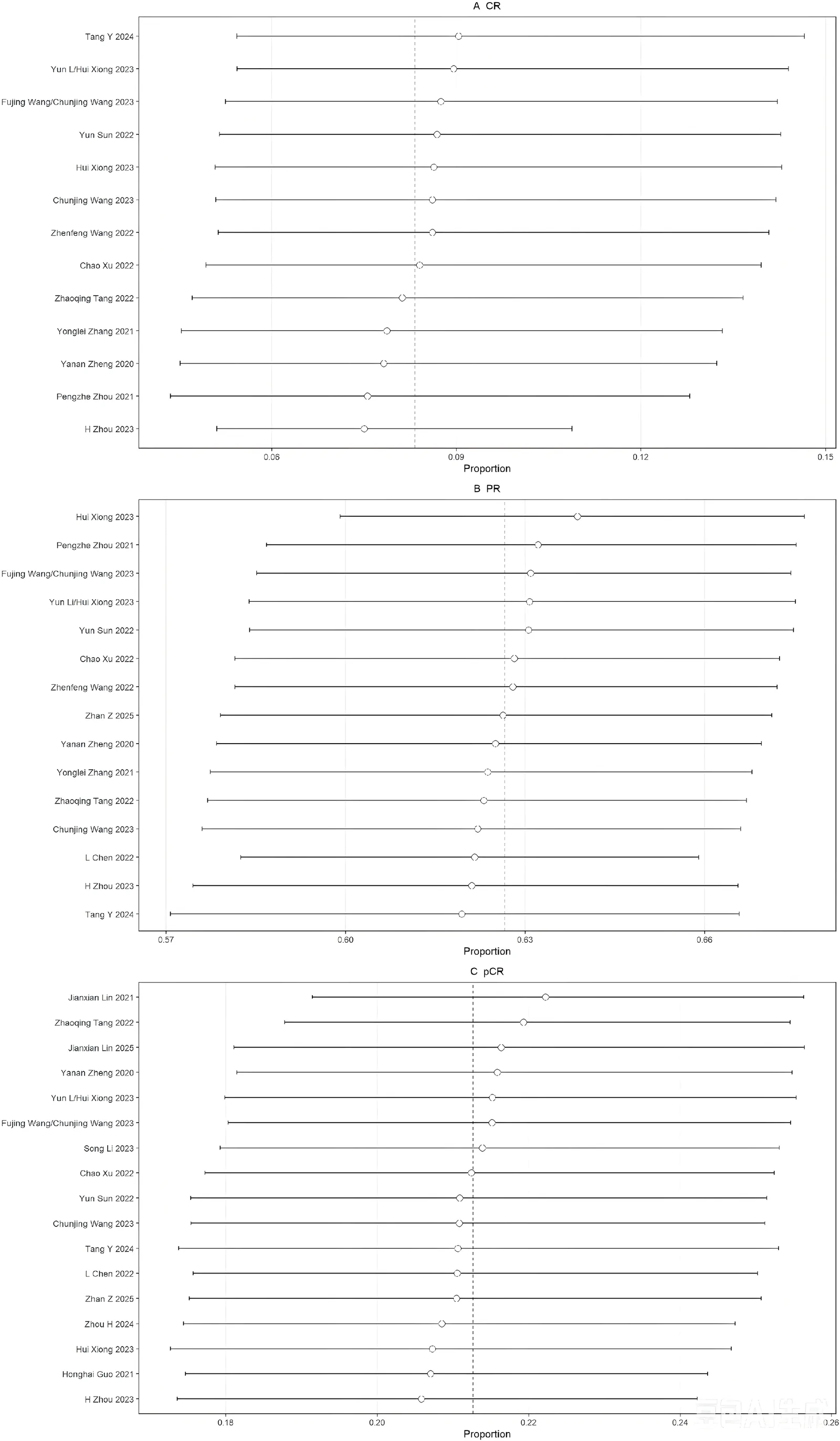
Sensitivity analyses for CR, PR, and pCR rates. Leave-one-out sensitivity analyses were conducted for **(A)** CR rate, **(B)** PR rate, and **(C)** pCR rate by sequentially omitting each included study. In each plot, the open circle represents the recalculated pooled estimate and the vertical bars represent the 95% confidence interval after removal of the indicated study. No substantial change in the pooled estimate or its confidence interval was observed after omission of any individual study, confirming the robustness of the results.

**Figure 10:**
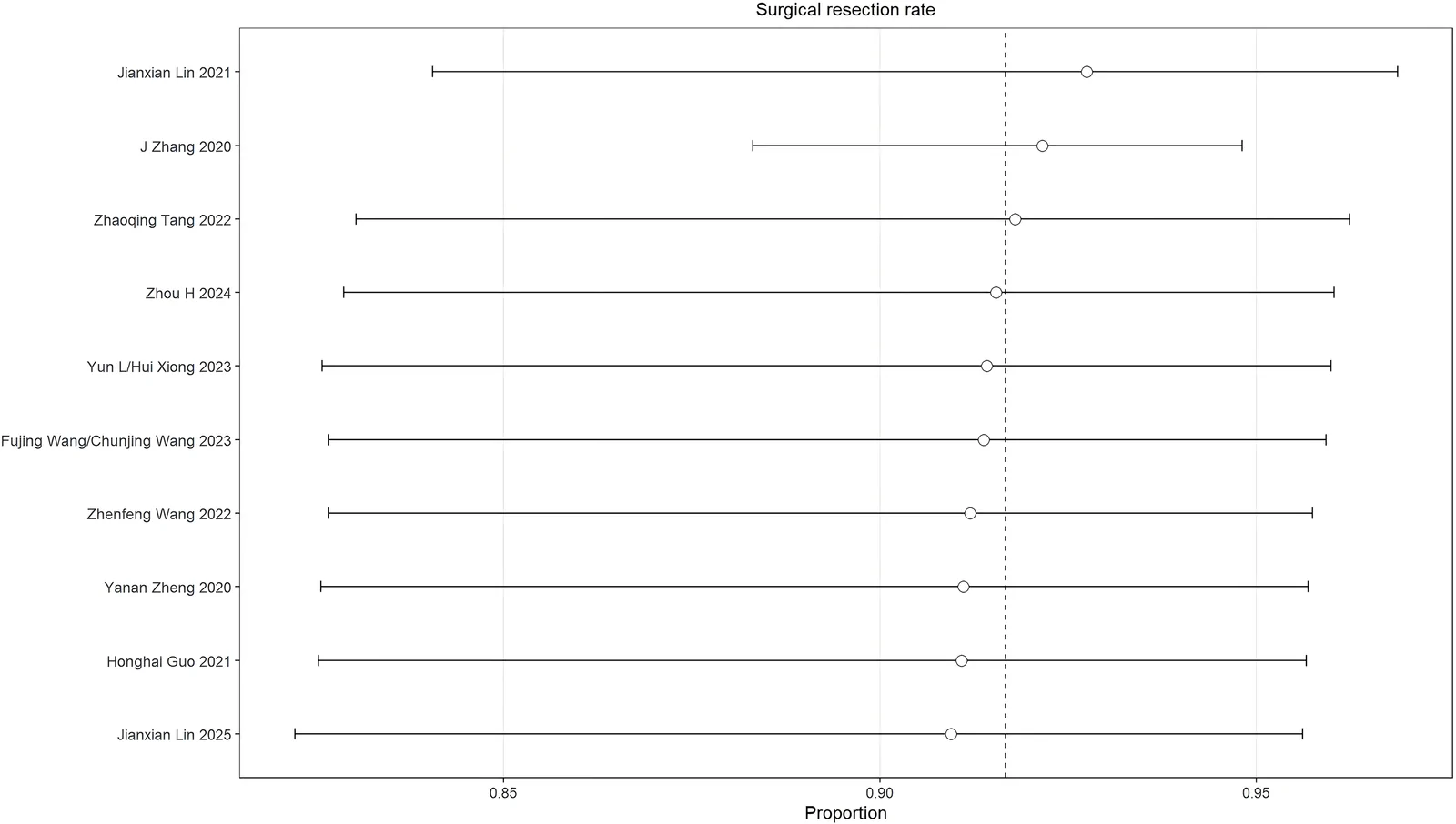
Sensitivity analysis for surgical resection rate. Leave-one-out sensitivity analysis for the pooled surgical resection rate. The open circles represent the recalculated pooled estimates and the vertical bars represent the 95% confidence intervals after sequential removal of each indicated study. The pooled estimates remained stable across all iterations, indicating that the pooled result was not unduly influenced by any single study.

#### Subgroup analysis

To further investigate potential sources of heterogeneity, subgroup analyses were conducted on neoadjuvant apatinib-based therapies. In descriptive subgroup analyses, separate pooled pCR proportions were 0.12 for nACT, 0.22 for nAICT, and 0.33 for nACRT (**Figure 11**). For MPR, CR, PR, and SD, separate descriptive pooled proportions by regimen are shown in **Figures 12**–**15**. No formal between-regimen statistical comparison was performed. Numerical differences among subgroups should not be interpreted as evidence of comparative efficacy.

**Figure 11:**
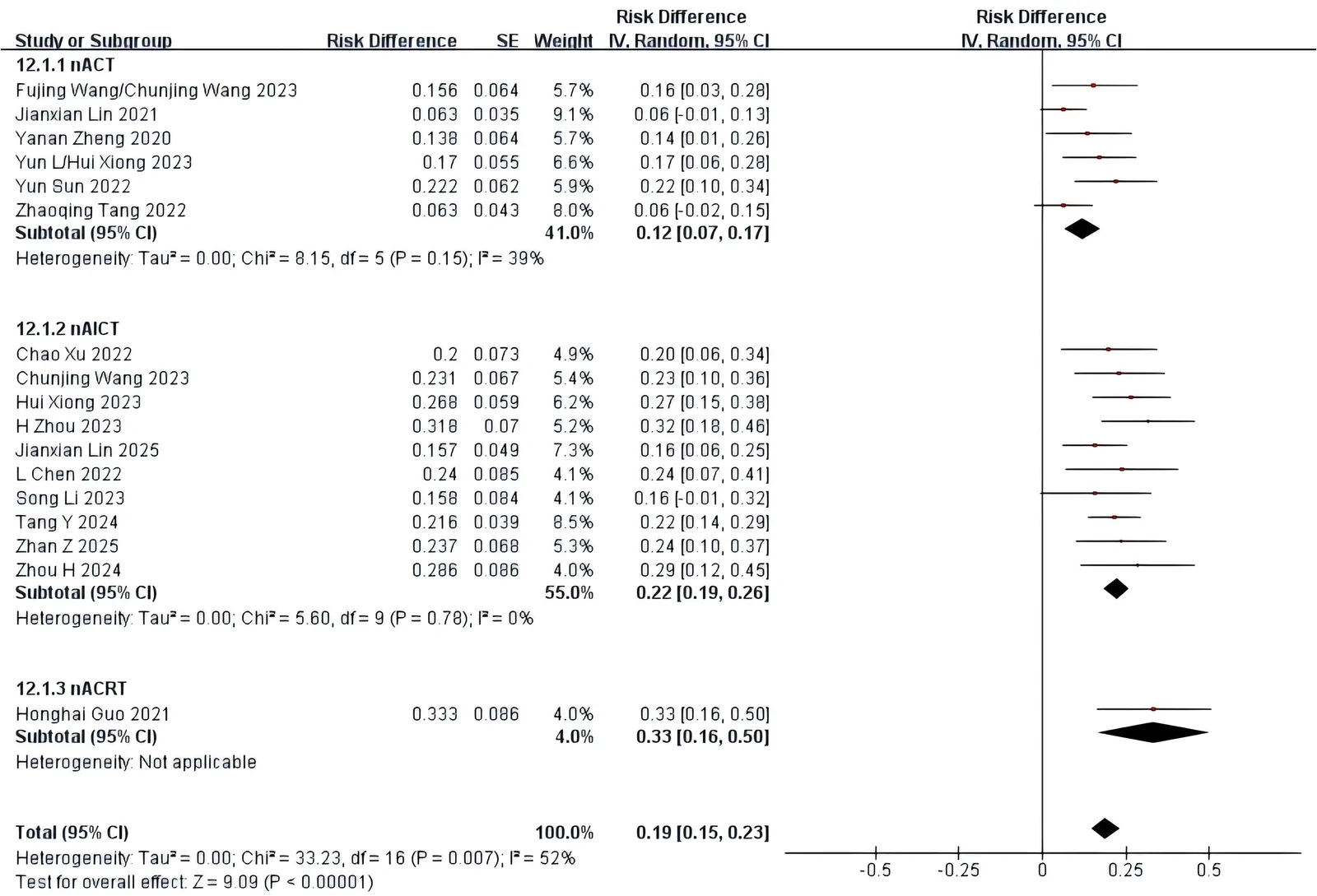
Subgroup analysis of pCR rates by treatment regimen. Forest plot comparing pCR rates across three neoadjuvant apatinib-based regimens: (12.1) nACT (apatinib + chemotherapy, 6 studies, pooled proportion = 0.12, 95% CI: 0.07–0.17), (12.2) nAICT (apatinib + immunotherapy + chemotherapy, 10 studies, pooled proportion = 0.22, 95% CI: 0.19–0.26), and (12.3) nACRT (apatinib + chemoradiotherapy, 1 study, pooled proportion = 0.33, 95% CI: 0.16–0.50). Each square represents the point estimate of an individual study, with horizontal lines indicating 95% confidence intervals; the diamond represents the pooled estimate within each subgroup. These are descriptive single-arm estimates; no formal between-subgroup comparison was performed, and numerical differences among regimens should not be interpreted as evidence of comparative efficacy.

**Figure 12:**
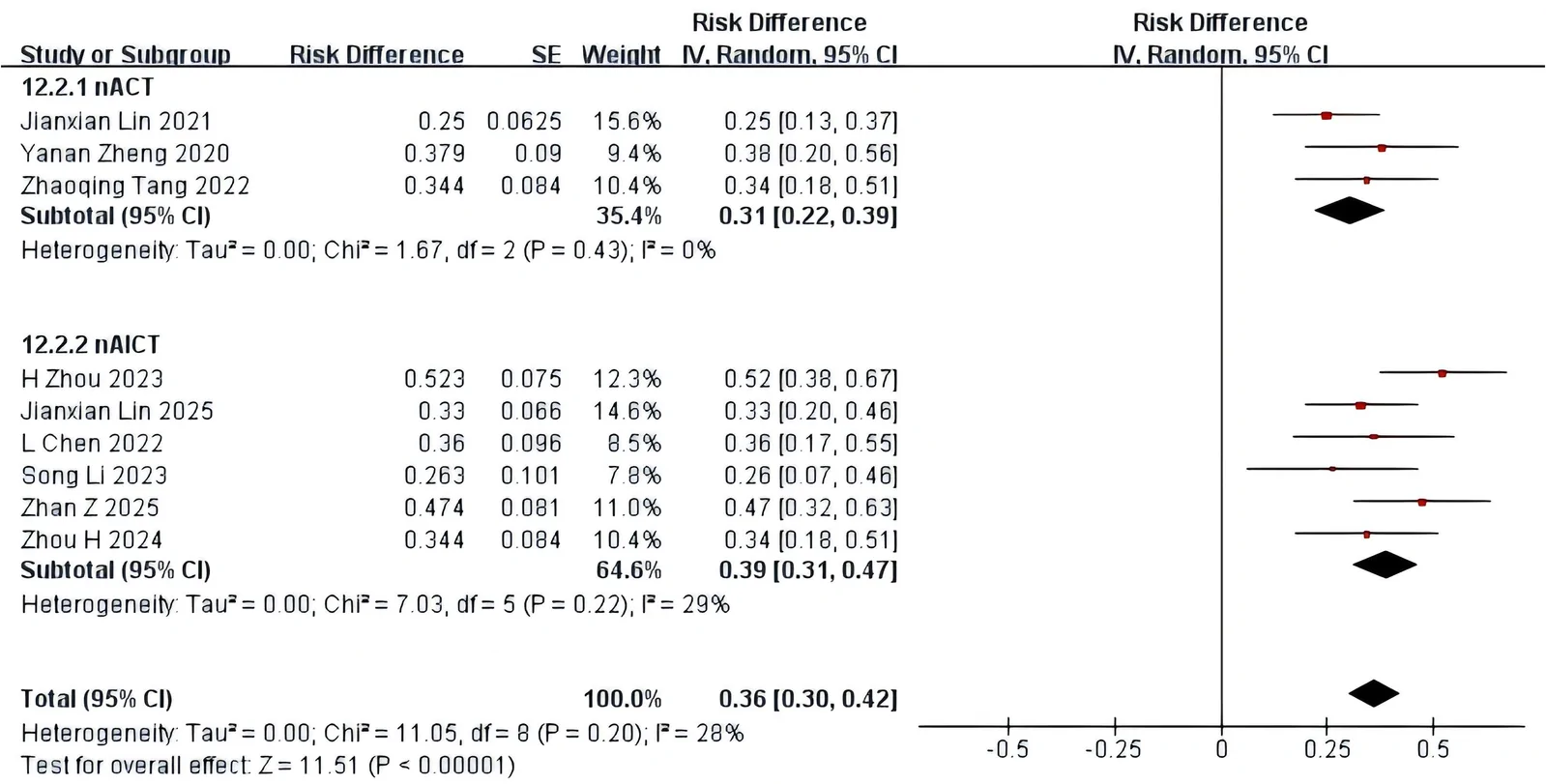
Subgroup analysis of MPR rates by treatment regimen. Forest plot of MPR rates by regimen: nACT (apatinib + chemotherapy, 3 studies, pooled proportion = 0.31, 95% CI: 0.22–0.39) and nAICT (apatinib + immunotherapy + chemotherapy, 6 studies, pooled proportion = 0.39, 95% CI: 0.33–0.46). These are descriptive single-arm estimates; no formal between-subgroup comparison was performed, and numerical differences between regimens should not be interpreted as evidence of comparative efficacy. Each square represents the point estimate of an individual study, with horizontal lines indicating 95% confidence intervals; the diamond represents the pooled estimate within each subgroup.

**Figure 13:**
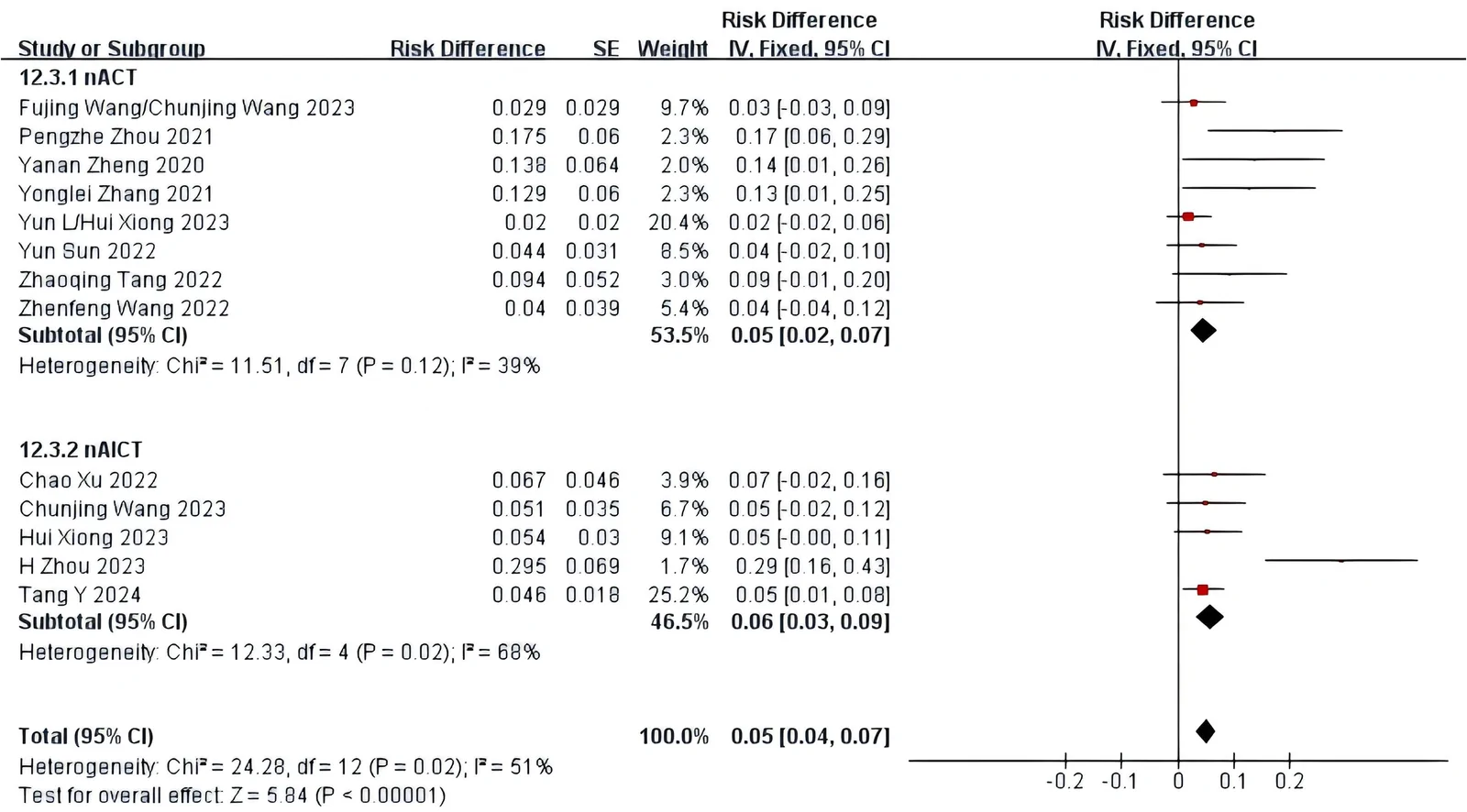
Subgroup analysis of CR rates by treatment regimen. Forest plot of CR rates by regimen: nACT (apatinib + chemotherapy, 8 studies, pooled proportion = 0.05, 95% CI: 0.02–0.07) and nAICT (apatinib + immunotherapy + chemotherapy, 6 studies, pooled proportion = 0.06, 95% CI: 0.03–0.09). These are descriptive single-arm estimates; no formal between-subgroup comparison was performed, and numerical differences between regimens should not be interpreted as evidence of comparative efficacy. Each square represents the point estimate of an individual study, with horizontal lines indicating 95% confidence intervals; the diamond represents the pooled estimate within each subgroup.

**Figure 14:**
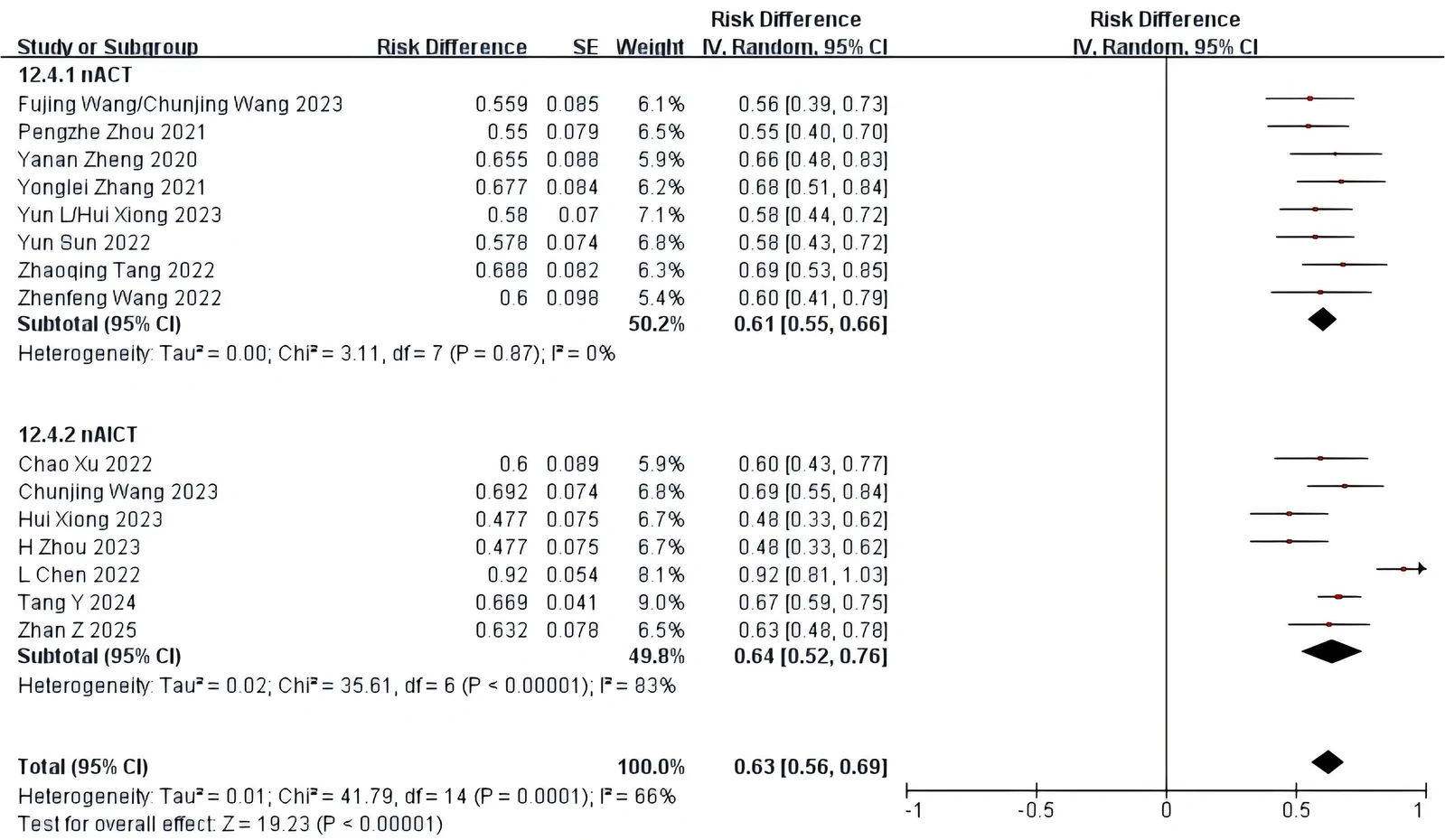
Subgroup analysis of PR rates by treatment regimen. Forest plot of PR rates by regimen: nACT (apatinib + chemotherapy, 8 studies, pooled proportion = 0.61, 95% CI: 0.55–0.66) and nAICT (apatinib + immunotherapy + chemotherapy, 8 studies, pooled proportion = 0.64, 95% CI: 0.52–0.76). These are descriptive single-arm estimates; no formal between-subgroup comparison was performed, and numerical differences between regimens should not be interpreted as evidence of comparative efficacy. Each square represents the point estimate of an individual study, with horizontal lines indicating 95% confidence intervals; the diamond represents the pooled estimate within each subgroup.

**Figure 15:**
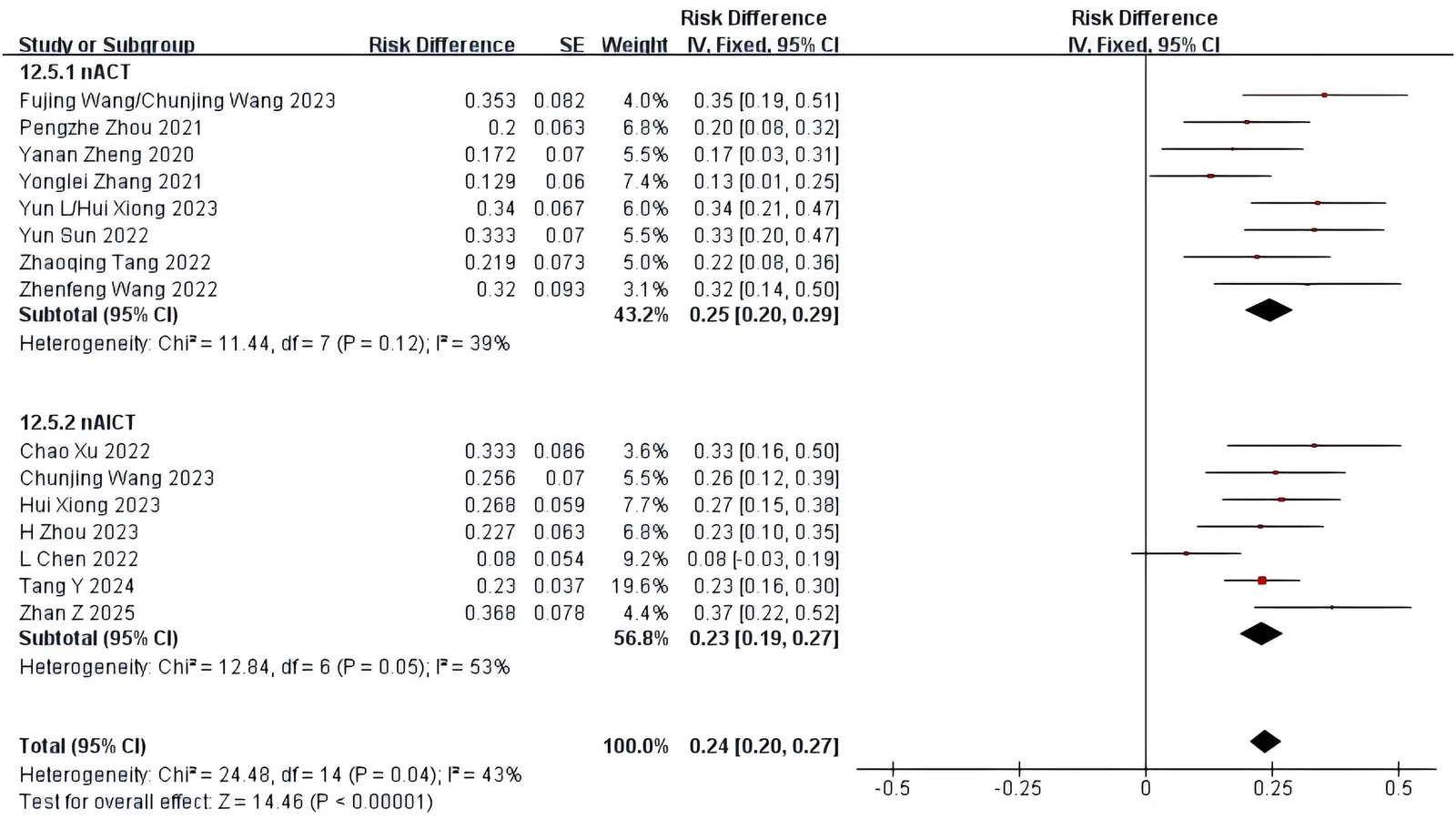
Subgroup analysis of SD rates by treatment regimen. Forest plot of SD rates by regimen: nACT (apatinib + chemotherapy, 8 studies, pooled proportion = 0.25, 95% CI: 0.20–0.29) and nAICT (apatinib + immunotherapy + chemotherapy, 8 studies, pooled proportion = 0.23, 95% CI: 0.19–0.27). These are descriptive single-arm estimates; no formal between-subgroup comparison was performed, and numerical differences between regimens should not be interpreted as evidence of comparative efficacy. Each square represents the point estimate of an individual study, with horizontal lines indicating 95% confidence intervals; the diamond represents the pooled estimate within each subgroup.

#### Publication bias

Publication bias was assessed using funnel plots for visual inspection (See the **Supplementary Image**), along with Egger’s regression test for quantitative evaluation. This analysis was conducted only for outcome measures that included ten or more studies. A *p*-value less than 0.05 in Egger’s test was considered indicative of statistically significant publication bias. Egger’s regression test did not reveal statistically significant publication bias for any outcome measure (all p > 0.05).

## Discussion

Despite the fact that neoadjuvant chemotherapy has been recommended by guidelines worldwide, the prognosis of patients with resectable G/GEJ remains suboptimal. The 5-year survival rates for patients with IIIA, IIIB, and IIIC were only 30.5%, 20.1%, and 8.3%, respectively (42). As far as we know, it is the first meta-analysis to evaluate the efficacy and safety of neoadjuvant apatinib therapy in patients with resectable G/GEJ. Our analysis is based on 19 small studies, including 975 patients, that quantitatively synthesized the efficacy and safety of neoadjuvant apatinib therapy. Because pathologic response to conventional neoadjuvant chemotherapy remains limited, regimens that can increase pathologic response while preserving complete resectability are being actively explored. The pooled pCR rate for neoadjuvant apatinib was 21%. In subgroup analyses, pCR rates were 12% in nACT, 22% in nAICT, and 33% in nACRT; however, the nACRT estimate was derived from a single study. The observed R0 resection rates were 93.5% in nACT and 96.7% in nAICT. These are descriptive findings from noncomparative single-arm studies and cannot be interpreted as evidence of improved response relative to conventional therapy. Subgroup analyses across different treatment regimens are exploratory in nature and not intended for confirmatory efficacy comparison; caution should be exercised when interpreting subgroup differences.

Descriptive pooled proportions of MPR, CR, PR, and R0 resection by regimen are shown in **Figures 12**–**15**. The pooled SD proportion was 0.25 (95% CI: 0.20–0.29) in nACT and 0.23 (95% CI: 0.19–0.27) in nAICT; these descriptive estimates were not formally compared. Regarding the pooled incidence of grade ≥r treatment-related adverse events (TRAEs), the pooled proportion was 0.31(95% CI: 0.21–0.43). The incidence was 0.23 (95% CI: 0.11–0.35) in the nAICT group and 0.39 (95% CI: 0.28–0.49) in the nACT group. The observed ORR was 70% in nACT and 74% in nAICT, and DCR was 94.8% in nACT and 95.4% in nAICT. These rates are reported descriptively and should not be interpreted as evidence of comparative efficacy relative to conventional therapy, as they derive from noncomparative single-arm studies without a concurrent control group. Prospective randomized controlled studies are needed for valid comparative assessment.

Grade ≥r TRAE proportions are reported separately for nAICT and nACT; the observed incidence was lower in the nAICT group than in the nACT group. High heterogeneity was observed, partly due to the inability of some studies to clearly distinguish between the number of patients and the number of events. Regarding the impact of neoadjuvant apatinib treatment on postoperative complications, the pooled results indicated an overall postoperative complication rate of 25.8% in the nAICT group (based on 4 studies), which was slightly higher than the rate of 22.2% observed in the nACT group (based on 4 studies). These descriptive findings should not be interpreted as evidence of a differential safety profile between regimens, and the current evidence does not permit firm conclusions.

In a three-arm randomized controlled study by Xiong and Li (41), it was reported that the nAICT group showed improved outcomes compared with the nACT group and the nAT group in terms of 1-, 2-, and 3-year DFS as well as 1-, 2-, and 3-year OS, meanwhile, the nAICT group could cause a higher incidence of peripheral neuropathy, and the nACT group could increase the incidence of hypertension in the patients. Possible explanations include PD-1 inhibitor-induced autoreactive antibodies, cytotoxic T cells, and inflammatory cytokines affecting neural integrity, as well as apatinib-induced endothelin-1 activation and oxidative stress (43, 44).

Angiogenesis plays a crucial role in tumor progression, with vascular endothelial growth factor (VEGF) and its receptors being key mediators of this process. Vascular endothelial growth factor receptor 2 (VEGFR2), one of the three VEGFR family members, exerts a pivotal effect on VEGF-mediated tumor angiogenesis. Apatinib is a novel anti-angiogenic agent that selectively targets VEGFR2 (45, 46). Apatinib also exerts its effect by targeting components of the tumor microenvironment (TME) outside tumor cells and promoting the infiltration and activation of CD8+ T lymphocytes (29). Clinically, immune checkpoint inhibitors targeting PD-1 have demonstrated antitumor activity in gastric and gastroesophageal junction adenocarcinoma (47), and apatinib-based regimens combined with PD-1 inhibition have shown antitumor activity in advanced gastric or gastroesophageal junction cancer and in other solid tumors (29, 48, 49). This dual anti-tumor mechanism not only directly exerts anti-angiogenic effects, but also lays a favorable foundation for the combination of apatinib with chemotherapy, immunotherapy and other anti-tumor therapies, making it a promising candidate drug for the multi-modal treatment of malignant tumors such as gastric and gastroesophageal junction adenocarcinoma.

Gastric adenocarcinoma is histologically heterogeneous, and in China, poorly differentiated adenocarcinoma, diffuse-type (Lauren) carcinoma, and signet-ring cell carcinoma are common. In our meta-analysis, the included studies generally did not stratify or report outcomes by histologic type or differentiation grade, and thus a reliable pooled subgroup analysis by histology/differentiation was not feasible. Available evidence on whether apatinib efficacy varies by histology or differentiation is currently limited and inconsistent: real-world studies of apatinib (e.g., the large AHEAD-G202 cohort (50)) enrolled both intestinal-type and diffuse-type tumors and did not demonstrate a clear, consistent difference in outcomes according to Lauren type or differentiation grade. Of note, poorly differentiated/diffuse-type tumors generally show lower pCR rates to conventional neoadjuvant chemotherapy; whether anti-angiogenic agents such as apatinib can specifically overcome this relative resistance remains unclear and warrants prospective investigation.

The most common adverse events associated with apatinib include hypertension, hand–foot skin reaction, proteinuria, fatigue, anorexia, and diarrhea, whereas myelosuppression and transaminase elevation are relatively uncommon. Bleeding, gastrointestinal perforation, and thromboembolic events, although rare, are serious adverse events that warrant particular attention. Tolerability may be further reduced in elderly patients. A phase II study specifically enrolling elderly patients with advanced/metastatic gastric cancer reported high incidences of fatigue (58.3%) and hypertension (47.9%) with apatinib (51). A sub-analysis of the large prospective real-world AHEAD-G202 cohort restricted to elderly (lde years) patients likewise showed that hypertension was the only grade 3–4 adverse event exceeding 5% (6.84%), with median overall survival and progression-free survival of 7.13 and 4.27 months, respectively (52). On the basis of this evidence, the NMPA-approved drug label in China advises that apatinib be used with caution in patients aged >70 years under medical supervision, with dose adjustment guided by clinical and laboratory findings. In addition, subgroup analyses of the REGARD and RAINBOW trials suggest that age does not appear to influence the efficacy of the VEGFR-2 monoclonal antibody ramucirumab (53), providing indirect support for continued evaluation of VEGFR-2 pathway inhibition in elderly patients. It should be noted that the median age of patients in the studies included in our meta-analysis was approximately 55–65 years, and none of the included studies provided age-stratified safety data; consequently, the pooled safety profile derived from our analysis may not fully represent very elderly patients (e.g., those aged ≥80 years). Accordingly, we recommend individualized management of elderly patients receiving apatinib-containing neoadjuvant therapy, including regular blood-pressure monitoring and standardized antihypertensive management, routine urinalysis to screen for proteinuria, early skin care and dose reduction when indicated for hand–foot skin reaction, and close monitoring of cardiac, renal, and coagulation function. For patients with poor tolerability or substantial comorbidities, a lower starting dose such as 250 mg/day may be considered.

### Limitations

Several limitations of this study should be fully acknowledged when interpreting the results. First, the overall level of evidence is relatively low, and the evidence base for quantitative synthesis is insufficient. The majority of included studies are single-arm phase II trials and retrospective cohort studies, with only a limited number of randomized controlled trials. The overall level of evidence from study designs is not high, with potential risks of selection bias and performance bias. In addition, several included studies are conference abstracts without full-text publication, which have not undergone complete peer review; the integrity of their data and methodological rigor need to be further confirmed by subsequent full-text publications. Pooling analysis based predominantly on low-level evidence inevitably limits the credibility of the overall conclusions. The results obtained are only exploratory and hypothesis-generating, and are insufficient to directly change clinical practice. Second, most included clinical trials have not yet reached their predefined long-term endpoints. The impact of neoadjuvant apatinib treatment on long-term survival outcomes such as overall survival and disease-free survival remains to be further confirmed by final follow-up data. Third, Substantial clinical heterogeneity existed across studies, including differences in apatinib dose, combination regimens, number of neoadjuvant cycles, tumor stage, histology, and patient selection. Methodological heterogeneity was also present, as most studies were single-arm, open-label, small-sample phase II trials or retrospective cohorts, and several were conference abstracts without full peer review. These factors limit the robustness of the pooled estimates and preclude definitive conclusions. Fourth, the influence of the number of neoadjuvant therapy cycles on key endpoints such as pCR rate remains unclear. Despite these limitations and heterogeneity, apatinib-based neoadjuvant treatment appears feasible with an acceptable safety profile in selected patients, and reports pathological response rates that may inform future hypothesis-driven research, providing a new direction for the future development of neoadjuvant therapy for resectable gastric/gastroesophageal junction adenocarcinoma.

## Conclusion

This meta-analysis synthesized the available evidence on neoadjuvant apatinib-containing regimens for resectable G/GEJ adenocarcinoma. The observed pooled pCR rate of 21%, MPR rate of 39%, R0 resection rate of 93%, and DCR of 95%, together with manageable grade ≥r TRAE rates (31%) and postoperative complication rates (27%), suggest that apatinib-containing neoadjuvant therapy is feasible in selected patients. However, as these descriptive estimates derive predominantly from noncomparative single-arm studies with substantial heterogeneity, no conclusion regarding comparative efficacy, tolerability, or benefit relative to conventional neoadjuvant therapy can be drawn. These observed rates provide a basis for the design of future large multicenter randomized controlled trials to validate the role of apatinib in the neoadjuvant setting.

## Data Availability

The original contributions presented in the study are included in the article/**Supplementary Material**. Further inquiries can be directed to the corresponding author.
