# Supplementary material for: Efficacy and safety of apatinib-containing neoadjuvant regimens for resectable gastric and gastroesophageal junction adenocarcinoma: a systematic review and single-arm meta-analysis
Source: Front Oncol. 2026 Oct 19;16:1910490. doi: 10.3389/fonc.2026.1910490 (PMC13724539; doi:10.3389/fonc.2026.1910490)
Supplement: Supplementary file 1 [file SupplementaryFile1.docx]

Supplementary form 1 Main features included in the studies

| Author  year | pCR | MPR | R0  resection rate | Surgical resection rate | Operative complication | TRG0 | TRG1 | TRG2 | TRG3 | ≥3  TRAEs |
| --- | --- | --- | --- | --- | --- | --- | --- | --- | --- | --- |
| Jian-Xian  Lin2021 | 3/48 | 12/48 | 36/40 | 40/48 | 7/38 | - | - | - | - | 16/48 |
| J. Zhang2020 | - | - | 19/20 | 20/33 | - | - | - | - | - | 11/33 |
| Yanan Zheng2020 | 4/29 | 11/29 | 28/28 | 28/29 | 12/28 | - | - | - | - | 10/29 |
| Yonglei Zhang2021 | - | - | 30/31 | 31/31 | - | 3/31 | 17/31 | 7/31 | 4/31 | - |
| Peng-zhe Zhou2021 | - | - | 31/40 | 40/40 | 7/40 | - | - | - | - | - |
| Zhaoqing Tang2022 | 2/32 | 11/32 | 31/32 | 32/35 | 7/32 | - | 16/32 | 13/32 | 2/32 | 18/32 |
| ZHENFENG WANG  2022 | - | - | 23/24 | 24/25 | - | - | - | - | - | - |
| Yun Sun  2022 | 10/45 | - | 43/45 | 45/45 | - | - | - | - | - | - |
| Fujing Wang/  Chunjing Wang2023 | 5/32 | - | 30/32 | 32/34 | - | - | 10/47 | 29/47 | 8/47 | - |
| Yun L/Hui Xiong2023 | 8/47 | - | 44/47 | 47/50 | - | - | 7/32 | 20/32 | 5/32 | - |
| L. Chen2022 | 6/25 | 9/25 | 25/25 | 25/25 | - | - | - | - | - | - |
| Song Li2023 | 3/19 | 5/19 | 19/23 | 23/23 | 7/20 | - | 5/19 | 4/19 | 10/19 | - |
| Chao Xu  2022 | 6/30 | - | 28/30 | 30/30 | - | - | - | - | - | - |
| H. Zhou2023 | 14/44 | 23/44 | 44/44 | 44/44 | - | - | - | - | - | - |
| Chunjing Wang2023 | 9/39 | - | 38/39 | 39/39 | - | - | - | - | - | - |
| Hui Xiong2023 | 15/56 | - | 53/56 | 56/56 | - | - | - | - | - | - |
| Lin, J. X. 2024 | 8/51 | 17/51 | 48/49 | 49/51 | 10/51 | - | - | - | - | 17/51 |
| Tang, Y. 2024 | 28/130 |  | 125/130 | 130/130 | - | 26/130 | 26/130 | 50/130 | 28/130 | 15/130 |
| Zhou, H. 2025 | 8/28 | 15/28 | 28/28 | 28/30 | 12/28 | - | - | - | - | 10/30 |
| Lin, J. X. 2025 | 8/51 | 17/51 | 48/49 | 49/51 | 10/51 | - | - | - | - | 22/51 |
| Zhan, Z. 2025 | 9/38 | 18/38 | 37/38 | 38/38 | 8/38 | - | - | - | - | 7/38 |
| Honghai Guo2021 | 10/30 | - | 28/30 | 29/30 | - | 10/30 | 6/30 | 9/30 | 3/30 | - |
