# Supplementary material for: Efficacy and safety of apatinib-containing neoadjuvant regimens for resectable gastric and gastroesophageal junction adenocarcinoma: a systematic review and single-arm meta-analysis
Source: Front Oncol. 2026 Oct 19;16:1910490. doi: 10.3389/fonc.2026.1910490 (PMC13724539; doi:10.3389/fonc.2026.1910490)
Supplement: Supplementary file 2 [file SupplementaryFile2.docx]

Supplementary form 2 Main features included in the studies

| Author  year | CR | PR | SD | ORR | DCR | 1 year -DFS | 2 year -DFS | 3 year -DFS | 1 year -OS | 2 year -OS | 3 year -OS |
| --- | --- | --- | --- | --- | --- | --- | --- | --- | --- | --- | --- |
| Jian-Xian  Lin2021 | - | - | - | - | - | - | - | - | - | - | - |
| J. Zhang2020 | - | - | - | - | - | - | - | - | - | - | - |
| Yanan Zheng2020 | 4/29 | 19/29 | 5/29 | 23/29 | 28/29 | - | - | - | - | - | - |
| Yonglei Zhang2021 | 4/31 | 21/31 | 4/31 | 25/31 | 29/31 | - | - | - | - | - | - |
| Peng-zhe Zhou2021 | 7/40 | 22/40 | 8/40 | 29/40 | 37/40 | - | - | - | - | - | - |
| Zhaoqing Tang2022 | 3/32 | 22/32 | 7/32 | 25/32 | 32/32 | - | - | - | - | - | - |
| ZHENFENG WANG  2022 | 1/25 | 15/25 | 8/25 | 16/25 | 24/25 | 91.7% | 75.2% | 75.2% | 100% | 89.6% | 78.4% |
| Yun Sun  2022 | 2/45 | 26/45 | 15/45 | 28/45 | 43/45 | 100% | 85.2% | 73.8% | 97.8% | 90.4% | 85.9% |
| Fujing Wang/  Chunjing Wang2023 | 1/34 | 19/34 | 12/34 | 20/34 | 32/34 | 86.1% | 72.8% | 49.9% | 96.9% | 76.7% | 70.3% |
| Yun L/Hui Xiong2023 | 1/50 | 29/50 | 17/50 | 30/50 | 47/50 | 86.9% | 75.3% | 63.7% | 94% | 79.5% | 76.1% |
| L. Chen2022 | - | 23/25 | 2/25 | 23/25 | 25/25 | - | - | - | - | - | - |
| Song Li2023 | - | - | - | - | - | - | - | - | - | - | - |
| Chao Xu  2022 | 2/30 | 18/30 | 10/30 | 20/30 | 30/30 | 96.60% | 77.70% | - | 96.60% | 90.10% | - |
| H. Zhou2023 | 13/44 | 21/44 | 10/44 | 34/44 | 44/44 | - | - | - | - | - | - |
| Chunjing Wang2023 | 2/39 | 27/39 | 10/39 | 29/39 | 39/39 | 100% | 85.7% | 76.1% | 100% | 96.3% | 86.7% |
| Hui Xiong2023 | 3/56 | 38/56 | 15/56 | 41/56 | 56/56 | 100% | 88.6% | 69% | 100% | 94% | 86.2% |
| Lin, J. X. 2024 | - | - | - | 33/51 | 49/51 | - | - | - | - | - | - |
| Tang, Y. 2024 | 6/130 | 87/130 | 30/130 | 93/130 | 123/130 | - | - | - | - | - | - |
| Zhou, H. 2025 |  |  |  | 24/28 | 28/28 | 92.6% | - | - | 92.4% | 84.4% | - |
| Lin, J. X. 2025 |  |  |  | 33/51 | 49/51 | - | - | - | - | - | - |
| Zhan, Z. 2025 | 0/38 | 24/38 | 14/38 | 24/38 | 38/38 | 94.7% | - | - | - | - | - |
| Honghai Guo2021 | - | - | - | 18/30 | 29/30 | 96.7% | 88.1% | - | 100% | 96.6 | - |
