# Supplementary material for: Efficacy and safety of apatinib-containing neoadjuvant regimens for resectable gastric and gastroesophageal junction adenocarcinoma: a systematic review and single-arm meta-analysis
Source: Front Oncol. 2026 Oct 19;16:1910490. doi: 10.3389/fonc.2026.1910490 (PMC13724539; doi:10.3389/fonc.2026.1910490)
Supplement: Supplementary file 3 [file SupplementaryFile3.docx]

| Study | A clearly  stated  aim | Inclusion of  consecutive  patients | Prospective  collection of  data | Endpoint  appropriate  to the study  aim | Unbiased  assessment  of endpoints | Follow-up  period  appropriate  to the major  endpoint | Loss to  follow  up not  exceeding  5% | Prospective  calculation  of the study  size | Total score |
| --- | --- | --- | --- | --- | --- | --- | --- | --- | --- |
| Jian-Xian  Lin2021 | 2 | 2 | 2 | 2 | 0 | 0 | 2 | 1 | 11 |
| Yanan Zheng2020 | 2 | 2 | 2 | 2 | 0 | 1 | 2 | 1 | 12 |
| Zhaoqing Tang2022 | 2 | 2 | 2 | 2 | 0 | 0 | 2 | 1 | 11 |
| L. Chen2022 | 2 | 2 | 2 | 2 | 0 | 1 | 2 | 1 | 12 |
| Song Li2023 | 2 | 2 | 2 | 2 | 0 | 2 | 2 | 0 | 12 |
| Chao Xu  2022 | 2 | 2 | 2 | 2 | 0 | 1 | 2 | 1 | 12 |
| H. Zhou2023 | 2 | 2 | 2 | 2 | 0 | 2 | 2 | 1 | 13 |
| Tang, Y2024 | 2 | 2 | 2 | 2 | 0 | 0 | 2 | 1 | 11 |
| Zhou, H2024 | 2 | 2 | 2 | 2 | 0 | 1 | 2 | 1 | 12 |
| Zhan, Z.2025 | 2 | 2 | 2 | 2 | 0 | 1 | 2 | 1 | 12 |
| Honghai Guo2021 | 22 | 2 | 2 | 2 | 0 | 2 | 2 | 1 | 13 |
