# Supplementary material for: Efficacy and safety of apatinib-containing neoadjuvant regimens for resectable gastric and gastroesophageal junction adenocarcinoma: a systematic review and single-arm meta-analysis
Source: Front Oncol. 2026 Oct 19;16:1910490. doi: 10.3389/fonc.2026.1910490 (PMC13724539; doi:10.3389/fonc.2026.1910490)
Supplement: Supplementary file 4 [file SupplementaryFile4.docx]

search strategies

Pubmed

("Stomach Neoplasms"[Mesh] OR "gastric cancer"[tiab] OR "stomach cancer"[tiab] OR "gastric adenocarcinoma"[tiab] OR "stomach neoplasm"[tiab] OR "stomach tumor"[tiab] OR "gastric tumor"[tiab] OR "gastric carcinoma"[tiab] OR "stomach carcinoma"[tiab] OR "gastric cardia cancer"[tiab] OR "cardiac cancer"[tiab] OR "gastroesophageal junction"[tiab] OR "esophagogastric junction"[tiab] OR "GEJ"[tiab] OR "esophagogastric junction adenocarcinoma"[tiab] OR "gastroesophageal junction adenocarcinoma"[tiab] OR "gastroesophageal junction cancer"[tiab] OR "esophagogastric junction cancer"[tiab] OR "Siewert"[tiab])

AND

("Apatinib"[Mesh] OR "apatinib"[tiab] OR "YN968D1"[tiab] OR "rivoceranib"[tiab])

AND

("Neoadjuvant Therapy"[Mesh] OR "neoadjuvant"[tiab] OR "preoperative"[tiab] OR "perioperative"[tiab] OR "before surgery"[tiab] OR "prior to surgery"[tiab] OR "neoadjuvant therapy"[tiab] OR "neoadjuvant treatment"[tiab] OR "preoperative therapy"[tiab] OR "preoperative treatment"[tiab])

Embase

('stomach cancer'/exp OR 'gastric cancer'/exp OR 'stomach tumor'/exp OR (gastric cancer OR stomach cancer OR gastric adenocarcinoma OR stomach neoplasm OR stomach tumor OR gastric tumor OR gastric carcinoma OR stomach carcinoma OR gastric cardia cancer OR cardiac cancer OR gastroesophageal junction OR esophagogastric junction OR GEJ OR esophagogastric junction adenocarcinoma OR gastroesophageal junction adenocarcinoma OR gastroesophageal junction cancer OR esophagogastric junction cancer OR Siewert OR 'Siewert type'):ti,ab,kw)

AND

('apatinib'/exp OR (apatinib OR YN968D1 OR rivoceranib):ti,ab,kw)

AND

('neoadjuvant therapy'/exp OR 'preoperative therapy'/exp OR 'perioperative therapy'/exp OR (neoadjuvant OR preoperative OR perioperative OR 'before surgery' OR 'prior to surgery' OR 'neoadjuvant therapy' OR 'neoadjuvant treatment' OR 'preoperative therapy' OR 'preoperative treatment'):ti,ab,kw)

Web of Science

TS=(gastric cancer OR "stomach cancer" OR "gastric adenocarcinoma" OR "stomach neoplasm" OR "stomach tumor" OR "gastric tumor" OR "gastric carcinoma" OR "stomach carcinoma" OR "gastric cardia cancer" OR "cardiac cancer" OR "gastroesophageal junction" OR "esophagogastric junction" OR GEJ OR "esophagogastric junction adenocarcinoma" OR "gastroesophageal junction adenocarcinoma" OR "gastroesophageal junction cancer" OR "esophagogastric junction cancer" OR Siewert OR "Siewert type")

AND

TS=(apatinib OR YN968D1 OR rivoceranib)

AND

TS=(neoadjuvant OR preoperative OR perioperative OR "before surgery" OR "prior to surgery" OR "neoadjuvant therapy" OR "neoadjuvant treatment" OR "preoperative therapy" OR "preoperative treatment")

Cochrane library

(MeSH descriptor: [Stomach Neoplasms] explode all trees OR (gastric cancer OR stomach cancer OR gastric adenocarcinoma OR stomach neoplasm OR stomach tumor OR gastric tumor OR gastric carcinoma OR stomach carcinoma OR gastric cardia cancer OR cardiac cancer OR gastroesophageal junction OR esophagogastric junction OR GEJ OR esophagogastric junction adenocarcinoma OR gastroesophageal junction adenocarcinoma OR gastroesophageal junction cancer OR esophagogastric junction cancer OR Siewert OR "Siewert type"):ti,ab,kw)

AND

(MeSH descriptor: [Apatinib] explode all trees OR (apatinib OR YN968D1 OR rivoceranib):ti,ab,kw)

AND

(MeSH descriptor: [Neoadjuvant Therapy] explode all trees OR (neoadjuvant OR preoperative OR perioperative OR "before surgery" OR "prior to surgery" OR "neoadjuvant therapy" OR "neoadjuvant treatment" OR "preoperative therapy" OR "preoperative treatment"):ti,ab,kw)
